# Nudging cooperation among agents in an experimental social network

**DOI:** 10.1007/s41109-023-00588-x

**Published:** 2023-09-12

**Authors:** Gorm Gruner Jensen, Martin Benedikt Busch, Marco Piovesan, Jan O. Haerter

**Affiliations:** 1 Niels Bohr Institute, University of Copenhagen, Blegdamsvej 17, 2100 Copenhagen, Denmark; 2 Department of Economics, Management, and Quantitative Methods (DEMM), University of Milan, Milan, Italy; 3 Department of Economics, University of Verona, Verona, Italy; 4 Center for Economic Behavior and Inequality (CEBI), University of Copenhagen, Copenhagen, Denmark; 5 Complexity and Climate, Leibniz Centre for Tropical Marine Research, Bremen, Germany; 6 Constructor University, Bremen, Germany

**Keywords:** Social network, Cooperation, Nudging

## Abstract

We investigate the development of cooperative behavior in networks over time. In our controlled laboratory experiment, subjects can cooperate by sending costly messages that contain valuable information for the receiver or other subjects in the network. Any message sent can increase the chance that subjects find the information they are looking for and consequently their profit. We find that cooperation emerges spontaneously and remains stable over time. In an additional treatment, we provide a non-binding suggestion about who to contact at the beginning of the experiment. We find that subjects partially follow our recommendation, and this increases their own and others’ profit. Despite the removal of suggestions, subjects build long-lasting relationships with the suggested contacts.

**Supplementary Information:**

The online version contains supplementary material available at 10.1007/s41109-023-00588-x.

## Highlights

- We investigate how humans interact in a network with no incentives to cooperate.
- Subjects build their own communication network and cooperation emerges spontaneously.
- A simple nudge about who and how many subjects to contact increases payoffs.
- Subjects form a higher-than-expected number of mutual relationships with others.

**Supplementary Information:**

The online version contains supplementary material available at 10.1007/s41109-023-00588-x.

## Introduction

Why do people cooperate, trust strangers, and build solid networks based on reciprocal help? This is one of the most fundamental puzzles in social sciences. Cooperation, trust and reciprocity are the key ingredients for the emergence of the so-called “social capital” that can foster economic and social development (Nowak and May 1992; Nowak and Sigmund 2005; Nowak 2006; Axelrod and Hamilton 1981). For instance, through cooperation, trust, and reciprocity, individuals can create and share information with one another to reach mutual help. The creation of *preferential communication networks* allows individuals to locate and access the exact information they need (Trusina et al. 2005; Sneppen et al. 2005) and sustain an *information flow* that is beneficial for the entire network (Rosvall and Sneppen 2003, 2006, 2009). This ability to exchange information is not trivial and cooperation is difficult to achieve in large networks that involve the coordination of many individuals simultaneously.

Under certain conditions, and often assuming simple games (Szabo and Fath 2007; Nowak et al. 2010; Tarnita et al. 2009), cooperation has been shown to emerge in both static (Rand, et al. 2014) and dynamic networks (Jordan et al. 2013). Such studies have led to the formulation of simple rules for when cooperation should rationally arise (Ohtsuki et al. 2006). Experimentally, findings may well depart from theoretical predictions, e.g., humans were found to maintain cooperation even when this would rationally not be expected (Grujic, et al. 2014), possibly due to moral preferences (Capraro and Perc 2021), although other works show that altruistic behavior may decline over time, e.g., under repeated games, to the benefit of rational deliberation (Gallotti and Grujić 2019).

The presence of time constraints (Fehl et al. 2011; Rand et al. 2011; Wang et al. 2012; Haerter et al. 2012; Bednarik et al. 2014; Bendtsen et al. 2016) together with the limited capacity of humans to focus on only a finite number of others (Fehl et al. 2011; Hill and Dunbar 2003; Miritello et al. 2013) are trademarks of human communication. Such features bring about a more complex game and make it hard to sustain an informal (non-binding) network of cooperation. In this paper, we study how humans interact, exchange information and learn to trust each other when such constraints are present. In a controlled and incentivized experimental setting, carried out using a sample of 100 human individuals in Copenhagen, Denmark, we observe subjects who freely build their own communication networks to find the information they need. We ask two questions: First, are subjects able to cooperate and create an efficient communication network even in a complex environment? Our data indicate that cooperation can emerge spontaneously and remain stable over time. Subjects create their own *preferential communication networks* that help them to achieve higher payoffs. Second, can a subtle and non-binding *nudge* foster cooperation and help sustain it over time? The provision of a weak suggestion about who and how many subjects to contact at the beginning of the experiment can foster communication and the stability of the network. Interestingly, the effect of the *nudge* survives long after its removal.

We create a controlled environment where the emergence of cooperation may be challenging: 25 players have to search for help in a large network for multiple rounds. At the beginning of each round, each player receives a unique *Question* and *Expertise* represented by neutral letters (e.g., *B* and *C*), where each player’s Question uniquely matches another player’s Expertise. To get a reward of 10 experimental currency units (ECU), players have to find out which of the other 24 players has the Expertise for their Question. Each round has two stages. In stage 1, players can send costly (1 ECU) inquiries to reveal their question and expertise to the receiver. For instance, the inquiry of a player who has Question B and Expertise C says: “I am an expert in C. I have a question about B. Can you help me?” Stage 1 ends with the simultaneous delivery of all inquiries. In stage 2, players can reply to the inquiries they received: each reply costs 1 ECU, and players cannot send false information. There are three types of replies depending on what information each player offers: (1) “I’m sorry, but I don’t know anyone who is an expert in B”; (2) “Yes! I happen to be the expert you are looking for”; (3) “The expert you are looking for is player X” if the player has received an inquiry from player X, that revealed that player X has expertise B. Stage 2 ends with the simultaneous delivery of all replies. Players can avoid sending an inquiry or a reply and their interaction remains private: no other player knows whether they helped each other or not. In our setting, players’ actions are only visible to those other players who interacted with them and, thus, they have the possibility to free ride without punishment.

In two separate sessions (nudging sessions), we gave players a visual suggestion for an efficient network structure during the first five rounds: a star appeared next to six players IDs, suggesting that each player send an inquiry to the six players marked with a star. We informed players that if every player follows our suggestion and helps other players find their expert, then all players will find their expert in every round. Note that in all our sessions, players can freely choose which players and how many to contact and whether or not to reply when others contact them. Note also that face-to-face communication is not allowed during the game.

The findings may have interesting implications for the structuring of human workgroups in firms, such that cooperation is fostered. Our study aims to explore a specific phenomenon by investigating the behavior of 100 students from the University of Copenhagen. We acknowledge that this sample size and narrow group of subjects may not fully represent the global population, and cultural, geographical, and sociodemographic factors can influence individuals’ responses in different contexts. Our study is intended as a pilot exploration, providing valuable insights into human cooperation, communication and trust building within its specific scope of professional networks. Additionally, to mitigate any potential bias, we took care of having a set of instructions, procedures, and frame to ensure an abstract and controlled environment of the experiment. We expect not to have induced any specific behavior or have confounded the main results with experimenter demand effects.

## Results

**Fig. 1:**
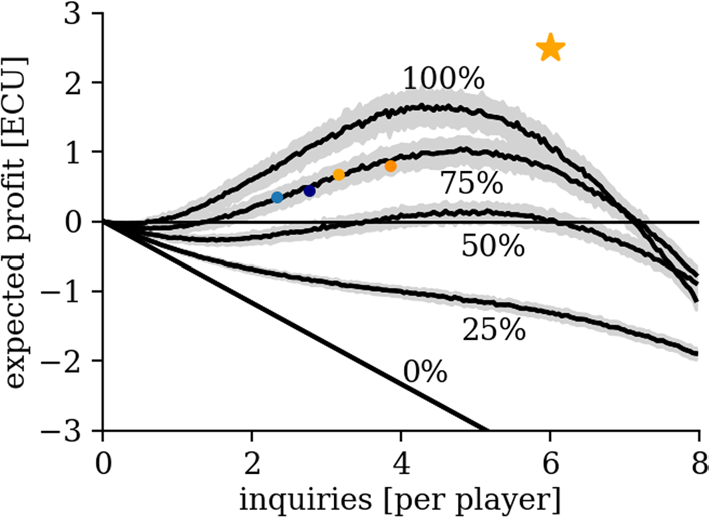
Expected Group Profit in ECUs. We generate an ensemble of 100 random networks for each number of total inquiries ranging from 0 to 8*24. Then we calculate the expected profit for each of the networks in the ensemble. The curves represent the mean of the ensembles, and the shaded areas represent the mean plus/minus one standard deviation. We calculate the expected profit by averaging over all possible question-expertise configurations for each network within an ensemble. We sum the profit of each player and take the average in each round to calculate the average group profit. We measure profit in Experimental Currency Units (ECUs, 1 ECU = 3 DKK). The colored dots (B1: light blue, B2: dark blue, N1: light orange, N2: dark orange) represent the four experimental sessions

**Fig. 2:**
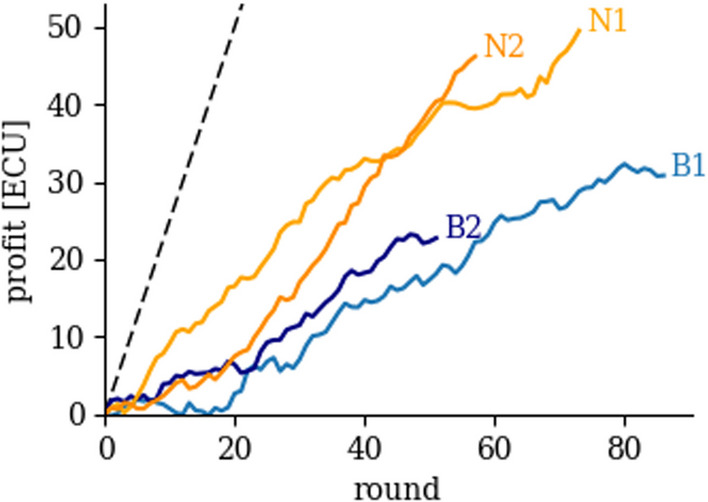
Average Profit (ECU) cumulated during each Session (B1, B2, N1 and N2). We sum the profit of each player and take the average in each round to calculate the average individual profit. We measure profit in Experimental Currency Units (ECUs, 1 ECU = 3 DKK). The number of rounds varies between sessions (86 rounds in B1, 51 in B2, 73 in N1, and 57 in N2). The dashed black line shows the expected profit if all subjects follow our suggestion

**Fig. 3:**
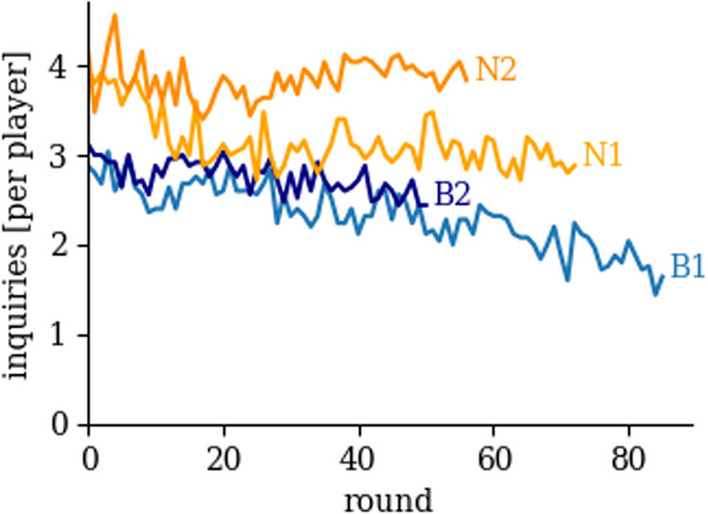
Average Cost for Sending Inquiries (in ECUs) per Session. We sum the number of inquiries of each player and take the average in each round to calculate the average cost. The number of rounds varies between sessions (86 rounds in B1, 51 in B2, 73 in N1 and 57 in N2). Note that the inquiry cost corresponds to the number of inquiries sent per player in a round

**Fig. 4:**
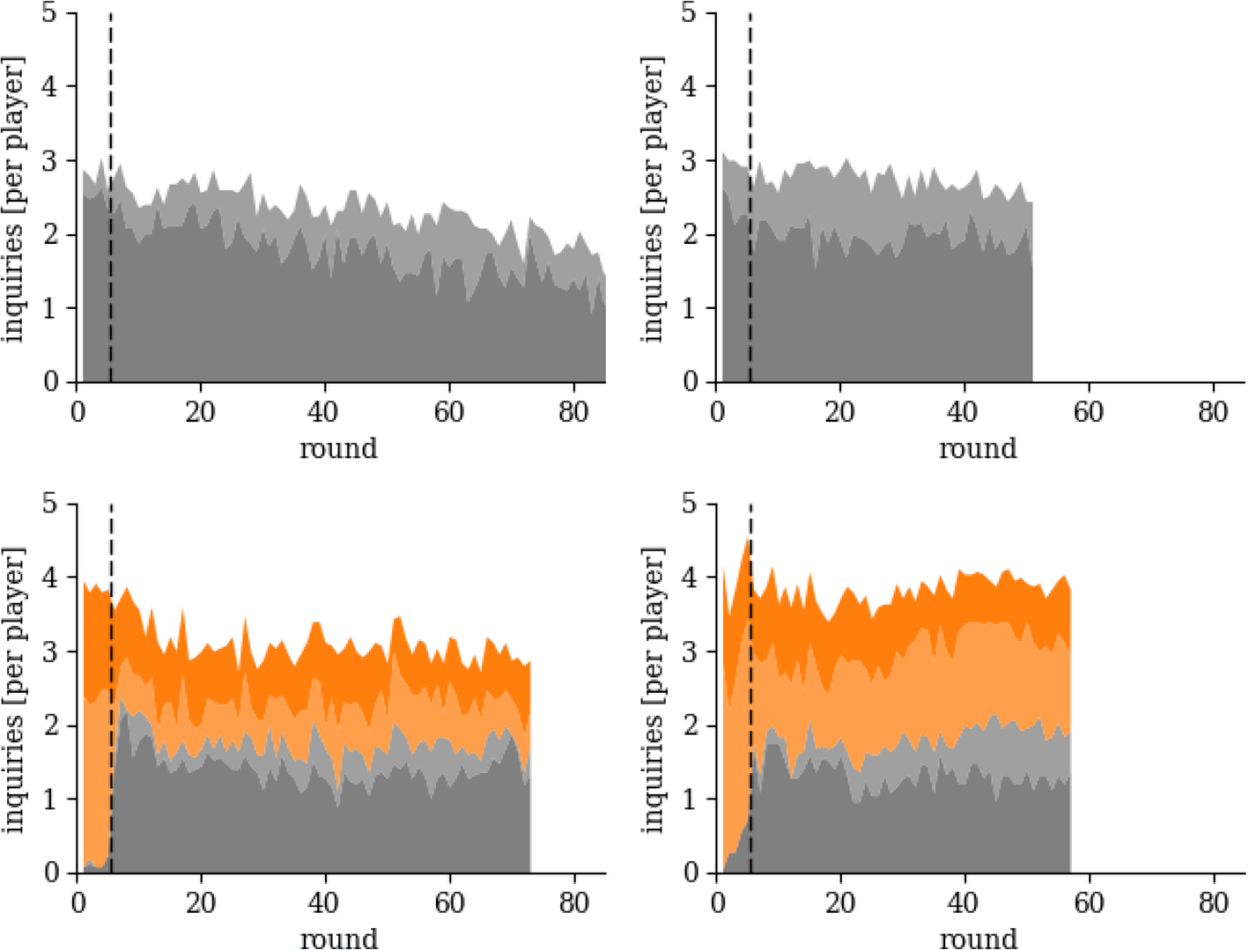
Decomposition of Inquiries Sent. The figure shows the number of inquiries sent split by the number of suggested links (light and dark orange) and non-suggested links (light and dark gray) in baseline and nudging sessions as a function of the number of rounds played. The lighter areas (both orange and gray) depict bi-directional links, whereas the darker areas (both orange and gray) depict unidirectional links. The dashed black line depicts the center between the last round in which suggestions are active (round 5) and the first round in which they are removed (round 6)

Our results show that subjects do cooperate: they are able to build reciprocal trust and sustain cooperation even if the incentives to do so are small (Figs. 1 and 2). Our simple nudge is effective in increasing the number of (costly) messages sent, leading to more information in the network and higher payoffs (Figs. 2 and 3). In the two baseline sessions (B1 and B2), subjects send inquiries even when others are not (fully) reciprocating. In the two nudging sessions (N1 and N2), subjects send even more inquiries: some inquiries following our suggestion, some to create and explore new networks (Fig. 4).

Figure 1 shows the expected profit as a function of the number of inquiries sent per player for random networks. We calculate the expected profit for a given number of inquiries and the reply rate, as follows. First, we calculate the probability that a player will find her expert by counting how many players know this and average this number over all combinations of experts. Second, we calculate how many replies a player will send, on average. Once we know the average number of messages (inquiries and replies) sent, it is straightforward to calculate the expected profit (see Additional file 1: S.3). When the reply rate equals zero, the expected profit is always negative. Players only find their experts when the expert contacts them through a direct inquiry. Therefore, expected profit linearly decreases with the number of inquiries sent. When the reply rate is greater than zero, one needs to take into account the probability that a player finds her expert through a third player to whom both the player and her expert sent an inquiry. For a reply rate of less than approximately 50%, the network does not generate a positive profit under the assumptions we made. First, we assume that every player only sends *informed replies*. An informed reply is a reply where player *i* sends a reply to an inquiry by player *j* containing the information on the requested expert. Second, we abstract from network structure (see Methods). The highest profit occurs when players send approximately $4.5$ inquiries, on average, given a reply rate of unity. The payoff structure of our game suggests that subjects should remain inactive unless they hold relatively strong beliefs about the number of inquiries their expert sends and others’ willingness to reply to inquiries (see Additional file 1: S.2). If all players follow our suggestion (orange star: efficient network structure of our suggestion), the expected profit increases compared to the curve that features a reply rate of 100%. The colored dots represent our four experimental sessions in which subjects made positive profits.

Figure 2 displays the (average) profits accumulated during the rounds played by 25 subjects in each of the four sessions (B1 and B2, in blue; N1 and N2, in orange). In the baseline sessions, our subjects do cooperate and earn, on average, 22.72 ECUs in B2 (min: − 35, max: 107) after round 51, and 80% of them can make a positive profit. In B1 subjects earned 18.12 ECUs after the same number of rounds (min − 45, max: 107), and 72% of them made a positive profit. We compare outcomes at round 51 as session B2 features the lowest number of rounds among all sessions. In the nudging sessions, subjects earned 39.12 ECUs in N1 (min: − 32, max 96) after round 51, and 84% of them can make a positive profit. In N2, subjects earned 40.36 ECUs (min: − 73; max: 105), and 88% of subjects made a positive profit. Across all rounds of a respective session (B1: 86, B2: 51, N1: 73, N2: 57) subjects make a profit of 30.8 ECU in B1 (min: − 39; max: 139), 22.72 in B2 (min: − 35; max: 107), 49.48 in N1 (min: − 40; max: 123), and 46.16 in N2 (min: − 54; max: 128). In total, 72% of subjects make a positive profit in B1, 80% in B2, and 84% in both N1 and N2. That is, average profit almost doubles (94.6% increase in profit at round 51) in nudging sessions as compared to baseline sessions. However, if subjects had perfectly followed our suggestion then accumulated profits in the nudging sessions would follow the dashed black line (see Additional file 1: S.5 for the derivation of expected profit for our suggestion).

Figure 3 shows the average number of inquiries sent per round during each of the four sessions. In the baseline sessions, our subjects cooperate by sending, on average, three inquiries in round 1 (B1: 2.88; B2: 3.12) and 2.65 inquiries per round across 51 rounds (B1: 2.53; B2: 2.76). In nudging sessions, subjects send, on average, about four inquiries in round 1 (N1: 3.96; N2: 4.16), which is lower than the six we suggested but significantly more than in baseline sessions (Wilcoxon Rank Sum test, two-sided, *p*-value = 0.00323, *n* = 50 in baseline and *n* = 50 in nudging). Note that observations are independent across all 100 subjects in the first round of the game. Across all 51 rounds, on average, subjects sent 3.53 inquiries (N1: 3.22; N2: 3.85). Comparisons of the number of inquiries sent per round across all sessions show that all session means differ significantly from each other at the 1% significance level (Pairwise Wilcoxon Rank-Sum test with bonferroni correction, *p*-value < 0.001, *n* = 51 per session). The increase in inquiries of 33.52% sent per subject (36.5% increase in inquiries and replies) doubles the cumulative profit even though the increase implies a higher cost.

There is a strong and positive correlation between how many inquiries subjects send and how many they receive (Pearson correlation coefficients [Inquiries sent – Inquiries received] = 0.73 (B1), 0.75 (B2). 0.51 (N1), and 0.60 (N2)). For the total number of messages sent (i.e., inquiries and replies), the sample Pearson correlation coefficients for each session are: B1: 0.84, B2: 0.9, N1: 0.73, and N2: 0.85. For more information, see Additional file 1: Figs. 3 and 4. In the post-experimental questionnaire, subjects reported that they interpreted an inquiry as a stronger friendship signal in the nudging compared to baseline sessions (two sample Wilcoxon rank sum test, two sided, *p*-value = 0.008). The share of replies sent is similar across sessions 0.82 (B1), 0.87 (B2), 0.85 (N1), and 0.81 (N2). To compute this rate, we divide the number of informed replies sent by the number of informed replies a subject could have sent. That is, subjects reply if they have the opportunity to do so, which implies that variation in the share of replies sent between baseline and nudging sessions cannot explain our results. In a post-experimental questionnaire, subjects said they valued received replies as a sign of “friendship” (the median subject gives replies a score of 9 out of 10 in baseline and nudging sessions) but most subjects (58% in baseline sessions and 60% in nudging sessions) stated that when sending replies, they did not distinguish between those they considered *friend*s (reply givers) and those they considered *acquaintances* (others not expected to give replies). Subjects sent only 3% of the possible *uninformative* replies (*I’m sorry, but I don’t know anyone who is an expert in X*). The percentage is similar across all sessions (B1; B2; N1; N2) = (1%; 6%; 2%; 4%). Subjects also sent redundant replies: for instance, if subject A is the expert that subject B is looking for, and they both send inquiries to each other, then it will be redundant for subject A to send a reply of the form “Yes, I’m the expert you are looking for”. Subjects sent these replies with a high frequency of ~ 75% (B1: 75%; B2: 77.4%; N1: 80.9%; N2: 67.7%), but subjects only had the opportunity to send such a reply about two times within 51 rounds. Hence, redundant replies cost only 5.44% of the profit in each session, on average. In the Additional file 1: S.3, we show how our measure of expected profit changes once we relax the assumption of informed replies by excluding redundant replies.

Our results indicate that the higher profits in nudging sessions can be explained by the higher number of messages (replies and inquiries) sent. However, our suggestion nudges subjects to send more inquiries as well as to send them along the suggested links. We disentangle the effect of the network structure from the number of inquiries sent and show that the number of inquiries sent drives the profit differences between baseline and nudging sessions rather than the network structure (see Additional file 1: S.1).

Figure 4 decomposes the number of inquiries subjects sent in N1 and N2 into “suggested links” (light and dark orange) and “others” (light and dark gray). In the initial five rounds during which the suggestions appeared on the screen, subjects sent almost all inquiries (96% in N1 and 91% N2) to a suggested link (light and dark orange). At round 6 we observe a sharp decline in the percentage of inquiries sent to suggested subjects, but after round 6 the share of inquiries sent to suggested subjects remains fairly constant (44% plus/minus 5% in N1 and 53% plus/minus 4% in N2). Thus, our initial suggestions influence network formation long after we removed them. We find that half of the reward is generated by interactions with suggested subjects (48% in N1 and 56% in N2). A subject receives a reward (10 ECU) if a message informs a subject about who their expert is. If a player receives more than one such message in a single round, then we divide the reward into equal fractions between subjects. However, as subjects do not perfectly follow our suggestion, we find that suggested links are slightly less efficient at generating a reward than links subjects form with others outside our suggestion. We calculate the Relative Return on Investment (RRI) for the first 51 rounds by summing the reward and dividing it by the number of inquiries (number of messages: inquiries and replies) sent as a measure of how much subjects get as a return from their investment in the respective group. In N1, the RRI per inquiry (per message) is 1.34 (1.14) among suggested links and 1.47 (1.27) among non-suggested links. In N2, the RRI per inquiry (per message) is 1.38 (1.14) among suggested links and 1.44 (1.21) among non-suggested links. In fact, suggested connections are bi-directional pairs of subjects simultaneously sending inquiries to each other, and thus are less efficient because of the redundancy of their information. We observe that there are more bi-directional links than we would expect to randomly occur in most of the rounds across all sessions (see Additional file 1: Fig. 5).

We separate bi-directional links (light orange and light gray) from unidirectional links (dark orange and dark gray) among suggested (orange) and non-suggested links (gray). Among suggested links in session N1 (N2), 61% (67%) are bi-directional in the first five rounds, on average. Whereas among non-suggested links, only 11% are bi-directional in session N1 in the first five rounds, on average. Not a single non-suggested link is bi-directional in N2 within the first five rounds. After round five, the percentage of bi-directional links among suggested links drops to 43% (58%) in N1 (N2), on average. Among non-suggested links, the percentage of bi-directional links increases to 19% (26%). In baseline sessions, the percentage of bi-directional links is 12% (18%) in B1 (B2) within the first five rounds. This number increases slightly to 24% (25%) in B1 (B2) after round five: subjects develop relationships with each other, leading to the formation of bi-directional links. In short, the share of bi-directional links among non-suggested ones is similar for baseline and nudging sessions. However, there is a large difference between sessions with respect to the share of bi-directional links due to our suggestion. In nudging sessions, the share of bi-directional links is not only larger, the large share of bi-directional links prevails at a constant level until the end of the experiment.

## Discussion

We studied the development of social capital in a controlled environment where subjects have little (or no) incentive to cooperate. We found that subjects are able to cooperate, trust each other, build reciprocal links and construct their own communication networks. Moreover, we demonstrated that a subtle and non-binding nudge is effective at fostering and sustaining cooperation even long after its removal. Our results indicate that the initial conditions and first interactions between subjects are crucial for shaping the information flow in self-organizing communication networks. Across all sessions, even within almost every round, subjects form a higher-than-expected number of bi-directional relationships with others. This happens despite the fact that bi-directional links lower the communication efficiency of the emergent communication network. The high frequency of bi-directional links therefore seems to suggest that human behavior is influenced by a desire to participate in reciprocal relationships.

Modern communication has become versatile, informal, and virtual, and has abandoned structured networks where links are pre-imposed. This type of communication has obvious advantages in terms of flexibility and it may be easier to use to accomplish creative or innovative tasks (Gallotti and Grujić 2019). Our experiment mimics virtual online communication where the success in exchanging information depends on previous interactions. An immediate gain is more attractive than the investment in a long-term relationship. Yet, mutual (or reciprocal) communication and strong links (Krackhardt et al. 2003; Melamed and Simpson 2016) facilitate the flow of information in email networks (Newman et al. 2002), in the world-wide-web (Albert et al. 1999), or Wikipedia (Zlatić et al. 2006)—hinting at the robustness of the finding of reciprocity for network links. However, the literature also suggests that weak ties promote organizational success (Friedkin 1982), as weak ties often allow for information flows across large societal distances (Granovetter 1977; Hansen 1999; Levin and Cross 2004).

Over longer timescales, emergent communication networks relate to the theory of social capital (Coleman 1988). The theory suggests that humans first build social capital by making investments and that they subsequently exploit social capital to achieve a benefit as a return. A theoretical mechanism leading to an emergent network structure can be optimization, where people make rational choices to garner personal benefits (Monge and Contractor 2001). However, the literature suggests that people “satisfice,” that is, they do not explore all options before settling for a good solution (Monge and Contractor 2001), often because it is simply impossible to do so. Further distorting the mathematical optimization problem, humans typically lack “time consistency,” and, thereby, they favor short-term gains that come with long-term losses (Gintis 2000).

Navigating the strategy space of a game as complex as ours in a purely rational manner would be an infeasible computational task for a human. So the subjects in our experiments likely acted more on intuition, rather than “solving” for the optimal solution. In our setting, we acknowledge that subjects suffer from optimization problems. Therefore, we tried nudging subjects towards a highly efficient solution. This initial nudging turned out to have a lingering effect on the emergent communication network, which resulted in a significant increase in average profits.

Our study focused on a specific group of participants, namely Danish students. As written above, we acknowledge that this sample may not be representative of the global population, and it aligns with the concerns raised regarding the focus of research on Western, Educated, Industrialized, Rich, and Democratic (WEIRD) populations (Henrich et al. 2010). It is crucial for future research to explore variations in behavior across more heterogeneous populations. For instance, our experimental protocol can be used with different subjects from other countries or backgrounds. By incorporating diverse samples and considering cultural, geographical, and sociodemographic factors, researchers can gain a more comprehensive understanding of how social capital can be built. Such investigations will provide valuable insights into the generalizability and robustness of our findings, paving the way for a more nuanced understanding of human behavior across different contexts.

That said, our results could have powerful practical implications. When building professional teams, such as in business or academia, initial “buddy programs” that kindly suggest professional partnerships between employees would establish informal contracts between them, thus, allowing the group to perform as an “information processing unit” long after the initial suggestion.

We constructed the “Suggested Network” that we implemented in nudging sessions from the perspective of a social planner who was interested in maximizing group profits while ensuring an equal distribution of profits among players. If we were solely interested in a network that maximizes group profits, we would have implemented a star network. However, in a star network, the central player would incur a large loss while everyone else would make a large profit. We did not do this as it would have been difficult to incentivize the central player to follow this strategy and we would not expect that this network would persist over time. We faced a trade-off between perfect equality and inequality. Social networks can provide a lens to understand the development and persistence of inequality in our society (Jackson 2021). A promising avenue for future research is to evaluate social networks as a source of inequality experimentally as the experimental tool allows the researcher to control the network features of interest.

## Methods

### Experimental data

We conducted a computerized experiment with 100 players shared in two baseline sessions (B1 and B2) and two nudging sessions (N1 and N2). We conducted the experiment at the Laboratory for Experimental Economics (LEE) at the University of Copenhagen, which approved that our study complies with the rules and regulations of the LEE. No subject participated in the experiment without informed consent. We acknowledge that this sample size may not fully represent the global population, and cultural, geographical, and sociodemographic factors can influence individuals’ responses in different contexts. Moreover, we recognize the limitations associated with studying a narrow group, as highlighted in the literature on WEIRD populations in social sciences (Jackson 2021). Our study is intended as a preliminary exploration, providing valuable insights within its specific scope. Additionally, to mitigate any potential bias, we took care of having a set of instructions, procedures and frame to ensure an abstract and controlled environment of the experiment. We expect not to have induced any specific behavior or have confounded the main results with experimenter demand effects.

In our game, we endowed players with 100 Experimental Currency Units (ECU; 1 ECU = 3 DKK) and, during the experiments, players made decisions that affected their earnings. Players earned 461.87 DKK on average (min: 71 DKK; max: 767 DKK) for approximately 180 min. We distributed written instructions about our interactive game to all players, and we checked their comprehension with on-screen control questions (see Additional file 1: S8). Our sessions have a different number of rounds (from 51 to 86) because we defined the game to last for exactly 90 min and the speed of each round depends on players’ behavior. In total, we observe *n* = 6675 decisions made by the 100 players across all sessions in stage 1 where players have to decide how many inquiries to send and to whom. Moreover, in stage 2, players send replies to the inquiries they received in the first stage. However, whether players are able to send a reply depends on whether they received an inquiry in stage 1.

Players’ ID numbers remain fixed throughout the session to allow each player to identify other players by their ID number, but otherwise players are anonymous. In practice, to a player sitting at computer *m*, the player sitting at computer *n* will appear with ID *n–m* mod 25. What varies at the beginning of each round is the unique *Question* and *Expertise* that each player receives from the computer, represented by neutral letters (e.g., *O* and *P*). Each player’s Question uniquely matches another player’s Expertise. First, we make a random permutation *p* of the 25 players and a random permutation *q* of the 25 first alphabet letters. We then assign to the player *p* the question *q* and the expertise *q* + *1* mod 25. In each round, players have to find out which of the other 24 players has the Expertise for their Question. If they succeed, they gain 10 ECU.

In nudging sessions, the six suggested IDs form a communication network with three properties:

1. The network is perfectly bi-directional (player *i* is suggested to player *j*, if and only if *j* is suggested to *i*), i.e., the adjacency matrix A is symmetric, $A_{ij} = A_{ji}$.
2. Each player is at most two links away from any other player, i.e., the network has diameter two.
3. The graph is symmetric in the sense that all nodes are topologically identical.

The Additional file 1: S.8 provides the detailed game instructions.

### The suggested network algorithm

We split the 25 players into five groups of five. Each player can now be indexed with two integers $g,i \in \left\{ {1,2,3,4,5} \right\}$, where *g* specifies which group the player is in and *i* is an index within the group. For a player with index $\left( {g,i} \right)$ we add the following six connections:

1. Link the groups together as a ring: $\left( {g, i + 1} \right)$ and $\left( {g, i - 1} \right)$.
2. Inter group links: ($$g + 1, 2i + 1$$), ($$g + 2, 3i + 3$$), ($$g + 3, 2i + 4$$), and ($$g + 4, 3i + 2)$$.

The indices should all be interpreted as modulo 5. That is, if an index is greater than 5 it loops around, for example 4 + 2 = 1 mod 5. It is clear that all players are treated symmetrically. It can be easily checked that the links are indeed bi-directional. Further, the network has diameter two, i.e., any two players are either directly connected or they have a common connection to whom they are both connected.

### Assumptions

The game derives its complexity from its network aspect and the trade-offs made by players as they choose who to communicate with. Classical game theory may not lead to straightforward results, as the size of the strategy space makes it difficult to even suggest the existence of a Nash equilibrium. As each player can send inquiries to any other within the first stage of each round, $2^{{n_{r} N\left( {N - 1} \right)}}$ combinations of inquiries are possible during a game comprising $n_{r}$ rounds and* N* players. Replies are conditional on inquiries, but further increase the options.

To describe basic game mechanics, we make the following two assumptions that simplify the strategy space. First, we assume that every player only sends *informed replies*. An informed reply is a reply where player *i* sends a reply to an inquiry by player *j* containing the information on the requested expert. We distinguish informed replies from *uninformed replies* of the form: “I’m sorry, but I don’t know anyone who is an expert in X”. Second, we abstract from network structure. We assume players can choose how many inquiries they want to send, but not who they send them to. We model the first round where players do not have a game history with each other, therefore, they do not have any incentive to prefer one player over another. The assumption implies that a selfish player does not have a rational incentive to reply to inquiries in the second stage. Without the incentive to reply, players do not have an incentive to send inquiries in stage 1. In this simplified version, standard rational agent theory predicts a single Nash equilibrium: Selfish players should remain inactive during the game and gain zero ECU. This is a typical example of the *tragedy of the commons* as replies increase group profit, but players have no incentive to send them. We define a reply rate *r* ∈ [0,1] capturing the probability that a player sends an informed reply. For example, if the reply rate equals unity, all players always send informed replies. If the reply rate is zero, players never send informed replies. For intermediate values, players will occasionally send informed replies.

### Ethics declaration

The experiment was conducted from October 2017 to April 2018. In accordance with Danish legislation at that time there was no need for an institutional review board approval (IRB) for this study, as sensitive data, as defined by the Danish Data Protection Agency, was not retrieved from subjects. The study was, however, revised and approved by the Center for Experimental Economics at the University of Copenhagen, which reviewed the process to ensure that the consent procedures, instructions, and data collection were conducted in line with good ethical research practice. When subjects choose to participate in an experiment after registration they give basic general consent that the University of Copenhagen can share the anonymized research data. Subjects volunteered to participate in our study. Subjects were introduced to the topic of the experiment on the first page of the instructions and could withdraw, at any time, if they did not want to participate. Subjects therefore provided their verbal informed consent to participate in the study. No written consent was obtained at this stage, as it is not required by the rules of the Center for Experimental Economics at the University of Copenhagen. The subjects signed a certificate of participation at completion of the experiment when paid. The certificate formally acknowledges their participation. All subjects signed the certificate.

### Supplementary Information

**Additional file 1:** Supplementary information containing technical details relevant to specialist readers but not needed at first reading of the manuscript.

## Data Availability

We have made data publicly available for researchers. It can be accessed at 10.5281/zenodo.8314798. Custom code that creates the figures and statistical analysis presented in this study can be accessed at 10.5281/zenodo.8314876.
