# Supplementary material for: Nudging cooperation among agents in an experimental social network
Source: Appl Netw Sci. 2023 Sep 12;8(1):62. doi: 10.1007/s41109-023-00588-x (PMC10497665; doi:10.1007/s41109-023-00588-x)
Supplement: Supplementary file 1 — Additional file 1: Supplementary information containing technical details relevant to specialist readers but not needed at first reading of the manuscript. [file 41109_2023_588_MOESM1_ESM.pdf]

Nudging cooperation among agents in an experimental social network

Gorm Gruner Jensen[1]

Martin Benedikt Busch[2,4]

Marco Piovesan[3,4]

Jan O. Haerter[1,5,6]

Supplementary Information

We thank Friederike Mengel, Antonio Cabrales, Nick Vikander, participants at the Workshop on Endogenous Institutions in Social Dilemmas and the Workshop on Experimental Economics meets Statistical Physics at the University of Copenhagen, and the Maastricht Behavioral and Experimental Economics Symposium for their helpful comments. The research activities of the Center for Economic Behavior and Inequality (CEBI) affiliates Marco Piovesan and Martin Benedikt Busch are funded by the Danish National Research Foundation through its grant (DNRF-134). Jan O. Haerter gratefully acknowledges funding by a grant from the VILLUM Foundation (grant number: 13168) and the European Research Council (ERC) under the European Union's Horizon 2020 research and innovation program (grant number: 771859). Corresponding author: Jan O.Haerter (haerter@nbi.ku.dk).

[1]Niels Bohr Institute, University of Copenhagen, Copenhagen, Denmark.

[2]Department of Economics, Management, and Quantitative Methods (DEMM), University of Milan, Milan, Italy.

[3]Department of Economics, University of Verona, Verona, Italy.

[4]Center for Economic Behavior and Inequality (CEBI), University of Copenhagen, Copenhagen, Denmark.

[5]Complexity and Climate, Leibniz Centre for Tropical Marine Research, Bremen, Germany.

[6]Constructor University, Bremen, Germany.

S Supplementary Information

S.1 Graph Structure and Sending Behavior

To explain the difference between baseline and nudging sessions, we study the effect of network structure and sending behavior on group profit. Recall that the suggested network induces an efficient network structure, but it simultaneously suggests subjects to send more inquiries. We use a measure of expected profit, which removes the effect of the inquiry network structure by re-shuffling of network links.

Supplementary Figure 1: Expected Group Profit in ECUs per Round

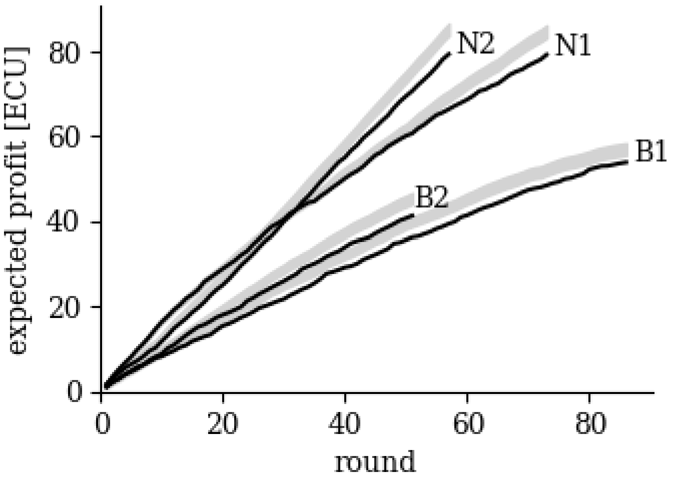

The figure shows the cumulated expected group profit averaged over all subjects within each session as a function of rounds (solid lines). The gray area shows the simulation outcome from 200 randomized game histories. The simulation preserves the number of inquiries sent by each subject in each round, but receivers are chosen at random with uniform probability. Each shaded area shows the mean plus/minus one standard deviation. We assume a reply rate equal to unity.

The expected profit is the mean profit that a player receives given the inquiry graph of a single round under the assumption that everybody sends informed replies with a reply rate equal to unity. We compute the average profit obtained by a player over all possible question/expertise configurations. The expected profit helps distinguish between the effects of the inquiry volume and network structure, because it allows us to directly compare the "efficiency" of the actual inquiry networks with randomized ensembles of the same inquiry volume.

The simulation works as follows. We preserve the number of inquiries sent in each round by each player, but the receivers are chosen at random with uniform probability. That is, this measure removes correlations due to relationships that players might form with each other over

time. Ideally, the network structure could help increase expected profit, as redundancy could be reduced relative to inquiries sent without coordination.

The opposite is the case for our experimental networks. The expected profit in any of the actual inquiry networks is consistently lower when compared to the ensemble where receivers are chosen at random with uniform probability. Whereas there are systematic differences in expected profits for the actual histories and the randomization, these differences are small compared to those between sessions. We conclude that it is predominantly the amount of sending behavior that is driving profit differences, not the network structure itself.

S.2 Expected profit when the expert sends *n* inquires

In the following, we show under which conditions a player should send at least one inquiry given other players' inquiry sending behavior and reply rate. We illustrate this using two representative players that we call Alice and Bob, where Alice is the expert for Bob's question. When should Bob send at least one inquiry? In other words, should Bob engage in playing the game at all? The answer depends on Bob's underlying beliefs about other players' behavior. First, it depends on whether Bob expects Alice to send inquiries and how many. Second, it depends on Bob's expectation about other players' likelihood of replying to inquiries. In the following, we derive belief thresholds under which Bob should engage in playing the game or not.

In a given round, consider the probability $P_{win}(m, n)$ that Bob finds Alice, where *m* denotes the number of inquiries Bob sends and *n* denotes the number of inquiries Alice sends. We assume that inquiries sent by Bob and Alice are sent to random receivers. Assume that Bob sends $m = 0$ inquiries. The probability that Bob finds Alice equates to $P_{win}(0, n) = n/24$. For example, if Alice sends $n = 1$ inquiry then this inquiry will reach Bob with probability $P_{win}(0, 1) = 1/24$ as there are 23 other players in the network. If Alice sends an inquiry to every other player in the network (that is, $n = 24$) then Bob will find Alice with certainty, that is, $P_{win}(0, 24) = 1$. We can denote the expected profit $\Pi_i(m, n)$ of Bob as follows:

$$\Pi_i(m, n) = 10 * P_{win}(m, n) - m.$$

The reward for finding Alice equates to 10 ECU, by definition. When Bob sends $m = 0$ inquiries, his expected profit equates to $\Pi_i(m, n) = \Pi_i(0, n) = \frac{10*n}{24}$.

Under which circumstances should Bob send at least m = 1 inquiry? To answer this, we need to calculate $\Pi_i(1, n)$ and compare it to $\Pi_i(0, n)$. Let us assume that the reply rate *r* equals unity in the following, so that players always reply if they can (we relax this assumption later). Note that once Bob sends an inquiry, there are two additional scenarios under which he can find Alice. First, Bob sends an inquiry directly to Alice. Second, Bob sends an inquiry to a player that Alice inquires too. To calculate the Probability $P_{win}(1, n)$ that Bob finds Alice, we can ask under which conditions Bob does not find Alice. This depends on the following two probabilities P_A and P_B:

$P_A = (24 - n)/24$: None of Alice's *n* inquiries are sent to Bob.

$P_B = (24 - (n + 1))/24$: Bob sends an inquiry to neither Alice nor any of the *n* players who Alice sends an inquiry to.

For example, if Alice sends one inquiry then the probability that the inquiry of Alice goes to any of the other 23 players equates to $P_A = 23/24$. In the other extreme, if Alice sends an inquiry to every player then Bob finds Alice with certainty, i.e., $P_A = 0$. Now consider the case where Bob and Alice send one inquiry each. The probability that Bob's inquiry reaches neither Alice nor any of the other players to whom Alice inquires equals $P_B = 22/24$. In the other extreme, if Alice sends *n*=23 inquiries, then Bob finds Alice with certainty, i.e., $P_B = 0$.

We can calculate the probability $P_{win}(1, n)$ that Bob finds Alice when Bob sends m=1 inquiry as follows:

$$P_{win}(1, n) = 1 - P_A * P_B = \frac{24+47*n-n^2}{24^2}. \quad (1)$$

Bob's expected profit equates to:

$$\Pi_i(1, n) = 10 * P_{win}(1, n) - 1 = \frac{240-24^2+470*n-10*n^2}{24^2}.$$

Thus, Bob should send an inquiry whenever $\Pi_i(1, n) \geq \Pi_i(0, n)$. We can solve this equation for *n*, yielding that $1.57 \leq n \leq 21.43$. Within this range of *n*, Bob should send at least one inquiry. Note that we derived this threshold under the assumption that the reply rate *r* equals unity. We can account for the reply rate being less than unity by considering the probability P_C as follows:

P_C: If Bob sends an inquiry directly to Alice or one of the *n* players to whom Alice inquired, Alice or one of the other players sends a reply with probability $r < 1$.

The probability P_C extends P_B as it is now possible that Bob does not find Alice even though he sends an inquiry directly to Alice, for example. We can denote the probability P_C by:

$$P_C = P_B + \left(1 - P_B\right) * (1 - r) = 1 - r * \frac{n+1}{24}.$$

Thus, when *r* equals unity, we recover $P_C = P_B$. However, if $r < 1$, the probability P_C exceeds P_B as players do not reply with certainty. When $r=0$ the probability P_C equals unity. Intuitively, if nobody replies, Bob cannot find Alice by sending inquiries himself. Substituting P_C for P_B in equation (1), the interval within which Bob should send at least one inquiry narrows. For example, the interval becomes $2.59 \leq n \leq 20.41$ $(5.09 \leq n \leq 17.91)$ for $r = 0.75$ $(r = 0.5)$. In other words, if Bob believes that other players do not always reply even though they know who the requested expert is, Alice needs to send more inquiries so that it is worthwhile for Bob to send at least one inquiry. Furthermore, we can calculate that when Bob believes that the reply rate is less than approximately 0.37 he should never send an inquiry independent of how few or many inquiries Alice sends. In Section S.4, we show that, for sufficiently high reply rates there exist non-trivial "active" Nash equilibrium states in addition to the "no-activity" Nash equilibrium.

S.3 Expected profit with random inquiry receivers

Assume a directed inquiry network I_{ij} describing a single round of the game, where $I_{ij} = 1$ if player i sends an inquiry to player j, and $I_{ij} = 0$, otherwise. We denote inquiry in-degree of player i by $I_i^{in} = \sum_j I_{ji}$ and inquiry out-degree of player i by $I_i^{out} = \sum_j I_{ij}$. If player j is player i's expert, then the number of players who can send informed replies to player i is:

$$x_{ij} = I_{ij} + \sum_k I_{ik} * I_{jk}.$$

The first term equals one when j can reply directly to player i and 0 otherwise. The second term adds one for every player k that receives an inquiry from player i and j. Player i wins the round if she receives a direct inquiry from player j, or by receiving at least one informed reply. Assume that there is a constant reply rate. Then the probability that player i does not receive any informed replies is given by $(1 - r)^{x_{ij}}$. The probability that player i finds her expert is given by:

$$P_{win}(i, j) = 1 - (1 - r)^{x_{ij}} * (1 - I_{ji}).$$

For example, player i is certain to find her expert (i.e., $P_{win}(i, j)$=1) if $I_{ji} = 1$, which means that her expert sends a direct inquiry to her so that $(1 - r)^{x_{ij}} * \left(1 - I_{ji}\right) = 0$. On the other hand, $P_{win}(i, j)$ solely depends on $(1 - r)^{x_{ij}}$ if player i's expert does not sent a direct inquiry (i.e., $I_{ji} = 0$). For example, if the reply rate equals zero, player i does not learn who her expert is with certainty (i.e., $P_{win}(i, j) = 0$). In the other extreme, when the reply rate equals unity, player i learns who her expert is with certainty given that $x_{ij} > 0$. All players have the same probability of being player i's expert, so the average probability of player i winning is:

$$P(w_i) = \sum_{j \neq i} P_{win}(i, j)/24.$$

The last thing we have to calculate is how many replies player i must send, on average. We distinguish two cases. In the first case, we calculate expected replies under the assumption that players send informed replies. We can write the expected number of replies $E\left(R_i\right)$ as follows:

$$E\left(R_i\right) = {I_i^{in}}^2/24.$$

For each inquiry player i receives, there is a $I_i^{in}/24$ chance that player i knows who the expert is. However, for bi-directional inquiry links, there is a 1/24 chance that player i is herself the expert that player j is looking for. In this case, player i knows that her reply does not contain any new information. Therefore, it would be more efficient for the group profit if player i refrains from replying. This would change the number of expected replies as follows:

$$E\left(R_i\right) = \left(I_i^{in} - I_i^{rec}\right) * \frac{I_i^{in}}{24} + I_i^{rec} * \frac{I_i^{in}-1}{24} = \frac{{I_i^{in}}^2 - I_i^{rec}}{24}.$$

The first term counts the number of non-reciprocal connections a player has with others times the probability that the player knows who the expert is. That is, the first term denotes the chance of knowing who the expert is given that the connections are non-reciprocal. The second term counts the number of reciprocal links a player has with others times the probability that the player knows who the expert is. Player i will reply to $r < 1$ of those, so that we can denote the expected profit of player i by:

$$\Pi_i = 10 * P_{win}(i, j) - \left(I_i^{out} + r * E(R_i)\right),$$

where the first term denotes the expected reward and the second term the expected cost consisting of the inquiry cost I_i^{out} and the expected reply cost $E\left(R_i\right)$. In essence, we can calculate

the probability that player i will win given that player j is her expert by counting how many players know this. We can average this number over each of the possible experts. In a similar fashion, we can calculate how many replies player i will be able to send on average. When we know the chance of winning and the average number of messages sent, it is straightforward to calculate expected profit.

S.4 Alternative Group Dynamics

Let us consider a different type of idealized game dynamics. We will investigate the expected profit of a player who sends m inquiries in a round when all other players send n inquiries each. The expected profit is the difference between the expected reward and expected cost (inquiry and reply cost). The expected reward is 10 ECUs times the probability of winning the round and the cost equates to 1 ECU for each message (inquiries and replies) sent:

$$\Pi_i(m, n) = 10 * P_{win}(m, n) - m - E(R_i).$$

First, we assume that all receivers are chosen independently with uniform probability. Second, all players send informed replies and a reply rate of unity. Given these assumptions we can calculate the probability of winning $P_{win}(m, n)$ and the expected number of informed replies $E(R_i)$ that player i sends:

$$P_{win}(m, n) = 1 - \frac{24-m-n}{24} \prod_{k=1}^{n} \frac{25-m-k}{25-k},$$

$$E(R_i) = \frac{n}{24} + 23 \cdot \left(\frac{n}{24}\right)^2.$$

The first term of $E(R_i)$ counts the expected number of direct replies to players that seek your expertise. The second term counts the number of replies where you can refer the expertise of players to each other. Supplementary Fig. 2 visualizes expected profits under the assumption of informed replies. One can see that the group can produce more profit, on average, if all players maintain a high level of sending activity (i.e., if they send many inquiries).

Supplementary Figure 2: Expected Profit of a Player when all Others' send *n* Inquiries

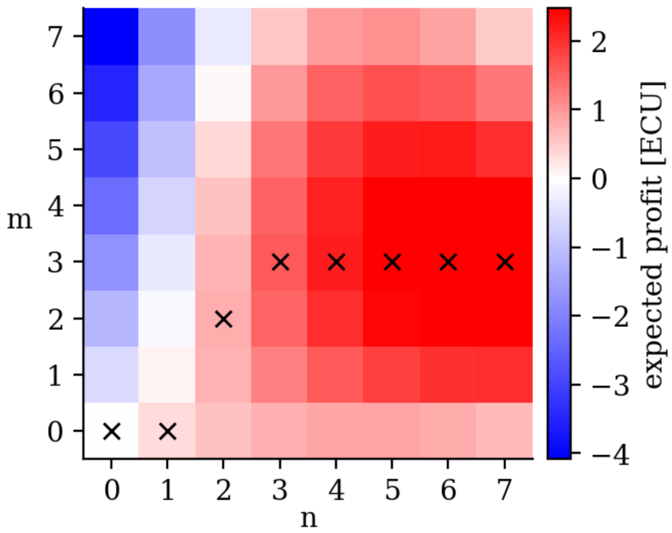

We sum the profit of each player and take the average in each round to calculate the average group profit. We measure profit in Experimental Currency Units (ECUs, 1 ECU = 3 DKK). The calculation of the expected payoff relies on two assumptions. First, all inquiry receivers are chosen at random from a uniform distribution. Second, all players send informed replies. The black x's indicate the optimal strategy for the players sending *m* inquiries, given all other players sending *n* inquiries.

However, players are trying to optimize their individual profit, rather than that of the group as a whole. What number of inquiries, m^*, will optimize a player's individual profit given that the other players all send n inquiries? In an environment where all other players send $n = 0$, n=2 or n=3 inquiries, all players can optimize their individual profits by conforming to the group and sending the same number of inquiries (i.e., $m^*=n$). These states can be interpreted as Nash equilibria of this restricted version of the game. In contrast, states where all players send $n>3$ inquiries each can be considered unstable, because the players have an incentive to reduce their sending activity. For n=4 or n=5 it is optimal to only send $m^*=3$ inquiries, so one could expect the overall activity to drop to this level. A similar logic applies for n=1, where every player would have an incentive to stop sending inquiries all together. Bringing the system into the completely silent state where all communication has perished.

The above considerations indicate that the game has a lower threshold for the overall inquiry activity below which it is no longer meaningful for the players to keep playing. The value of the threshold might very well be different in the actual game, given that we have ignored the effects of non-random network structures. We also assume that all players except one

send the same number of inquiries. However, we can make the qualitative prediction that the inquiry activity should start to consistently decay once it gets below some threshold.

Note that replies stemming from the first term do not contain any new information if a player already sent an inquiry. This happens with a probability of $m/24$, which changes the average number of replies as follows:

$$E(R_i) = \frac{n}{24} \cdot (1 - \frac{m}{24}) + 23 \cdot (\frac{n}{24})^2.$$

S.5 Expected Profit of the Suggested Network

We provide a suggestion to subjects to create a situation where it is entirely up to themselves to maintain an efficient network. Our suggestion maximizes the expected profits for each subject, while ensuring an equal distribution of profits among subjects. The network we suggest is bi-directional (i.e., all links are reciprocal) and yields the same expected profits for each player (i.e., perfect equality) as opposed to a network that maximizes expected profits (e.g., perfect inequality in a star network). The network connects all 25 players such that each player receives the same expected profit independent of her position in the network. The local network (first degree neighborhood) of each position exhibits the same properties, so that each position receives the same profit in expectation.

To calculate the expected profit $\Pi_i(m, n)$ for each player in the suggested network within a given round, we make the following two assumptions. First, players perfectly follow our suggestion in stage 1 of each round, which implies that each player sends the same number of inquiries, $\Pi_i(m, n) = \Pi_i(m, m) = \Pi_i(m)$. Second, we assume a reply rate of unity and that players only send informed replies in stage 2 of each round. Note, the two assumptions ensure that every player finds her expert in every round. Given that a player gains 10 ECUs if she finds her expert, the expected reward per player equates to 10 ECUs per round.

We calculate the expected cost of sending inquiries and replies for a given round. Calculating the expected cost of sending inquiries is straightforward as 25 players send six inquiries each round according to our suggestions. Each inquiry costs 1 ECU, hence the expected cost of sending inquiries equates to six ECUs per player. We calculate the expected reply cost

$E(R_i(m))$ as follows. For each inquiry a player receives, the probability $P_R(m)$ that the requested expert is one of the players she is linked to equates to:

$$P_R(m) = \frac{m}{N-1},$$

using our definition of informed replies where *N* denotes the total number of players. However, for bi-directional inquiry links, there is a 1/24 chance that player i is the expert herself, in which case the reply does not contain any new information. We can account for this and rewrite the probability as follows:

$$P_R(m) = \frac{m-1}{N-1}.$$

Note that it is $m - 1$ because a player cannot send a reply to someone who is already linked to her. Remember that when the link is bi-directional, the knowledge about question and expertise pairs is already transferred through inquiries and part of the expected cost of sending inquiries. This being the case, we only have to take the probability into account that one has to refer her direct links to each other. Putting everything together, the expected reply cost for each player at any given round is:

$$E(R_i(m)) = P_R(m) * m.$$

The final step is to insert the parameters specific to the game. The experiment features *N*=25 players that send six inquiries to each of the six suggested players. This yields expected reply costs of $E(R_i(m)) = 1.5$ ECUs per player and round using the assumption of informed replies. The expected profit $\Pi_i(m)$ of each player, therefore, equates to 2.5 ECUs per round. Lastly, we allow for the possibility that a reply sent along a bi-directional link might not contain any new information. In this case, expected reply costs equate to $R_i(m) = 1.25$ ECUs per player, which yields an expected profit of $\Pi_i(m) = 2.75$ ECUs per player and round.

S.6 Additional Figures

Supplementary Figure 3: Reciprocal Inquiry Sending Behavior

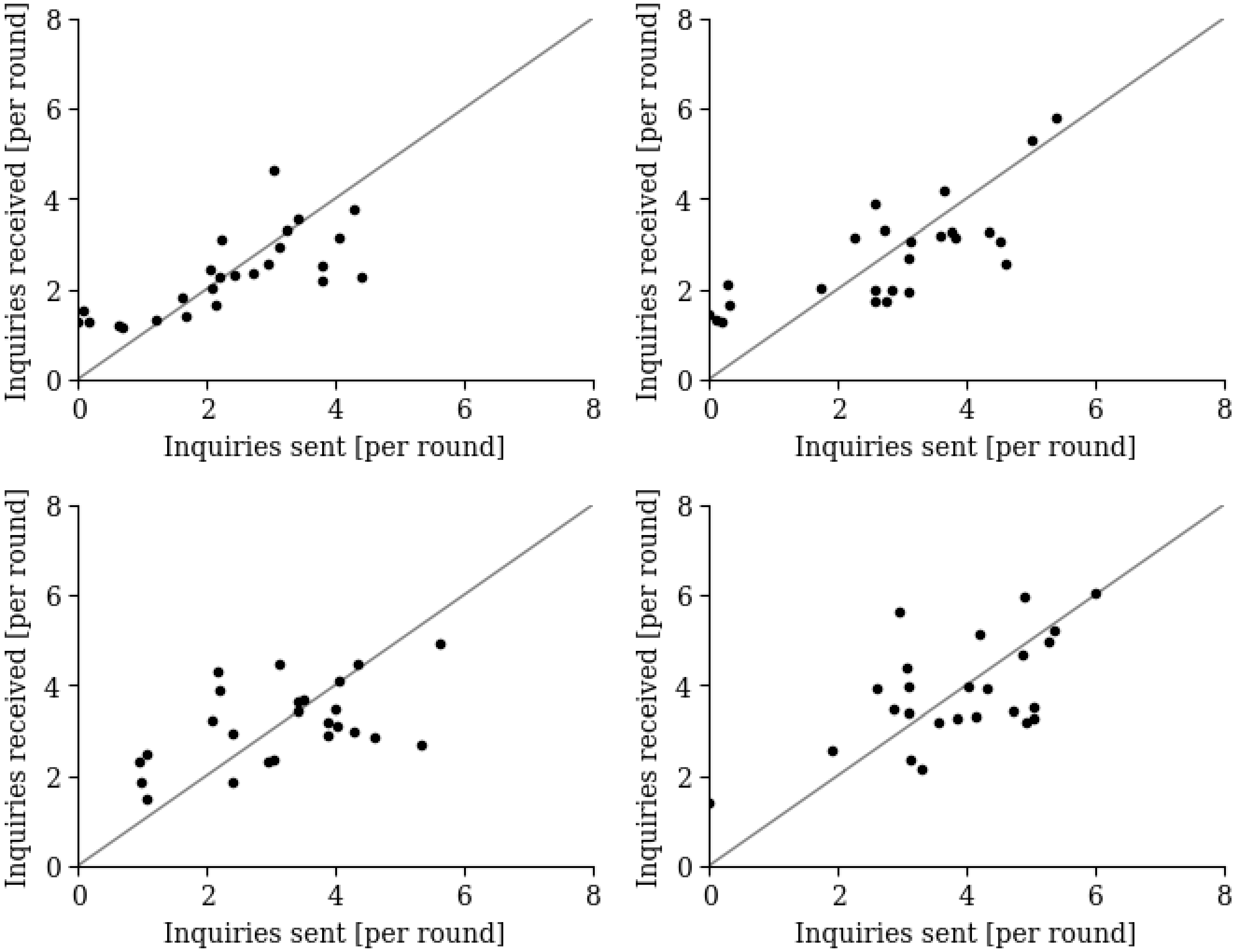

The scatter plots show the number of inquiries received as a function of the number of inquiries sent, normalized by the number of rounds played in the respective session. The number of rounds varies between sessions (86 rounds in B1, 51 in B2, 73 in N1, and 57 in N2). Each dot represents one out of 25 players for baseline sessions (B1 and B2) and nudging sessions (N1 and N2). The black line denotes the 45-degree line.

Supplementary Figure 4: Reciprocal Message Sending Behavior

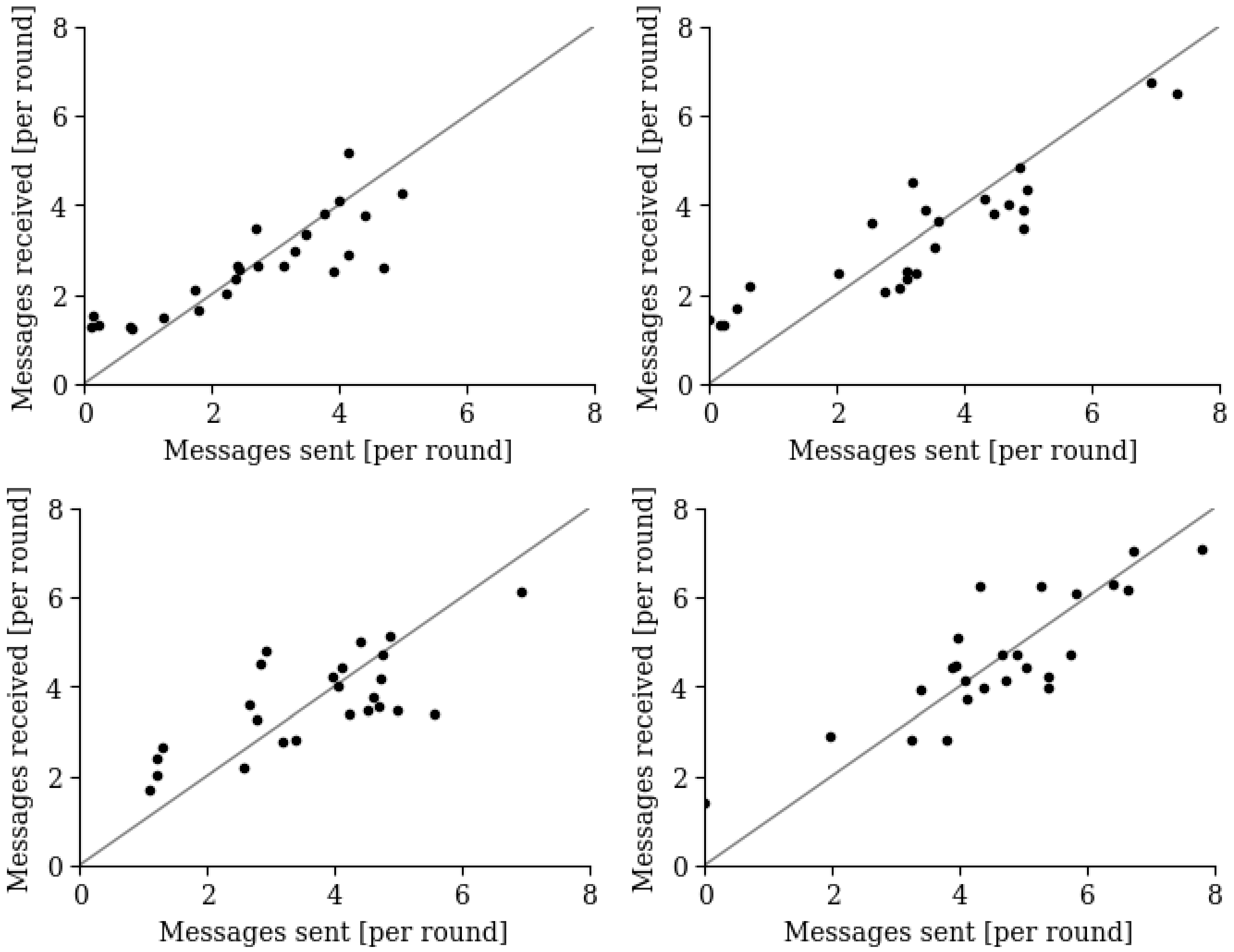

The scatter plots show the number of messages received as a function of the number of messages sent, normalized by the number of rounds played in the respective session. The number of rounds varies between sessions (86 rounds in B1, 51 in B2, 73 in N1, and 57 in N2). Each dot represents one out of 25 players for baseline sessions (B1 and B2) and nudging sessions (N1 and N2). The black line denotes the 45-degree line.

Supplementary Figure 5: Statistical Over-Representation of Bi-directional links

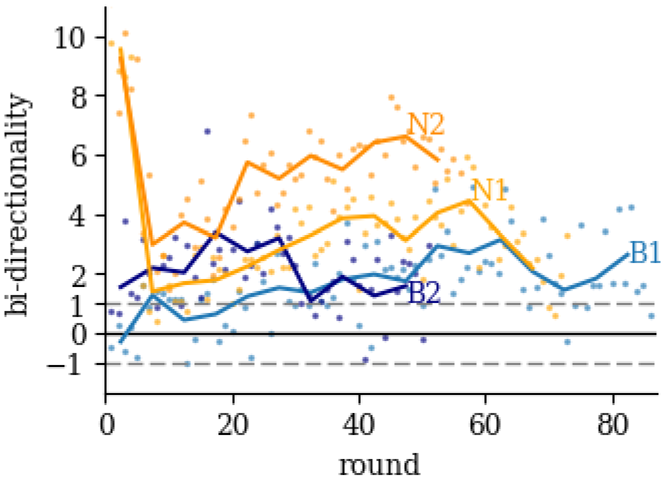

We generate an ensemble of 100 random inquiry networks for each round in each session, preserving the in- and out-degrees of the actual (data) networks. We measure the mean and standard deviation of bi-directional links in the ensembles - again for each round in each session. The y-axis shows the standard deviation of our normalized bi-directionality measure. We construct this measure by subtracting the ensemble mean from the actual number of bi-directional links and divide by the standard deviation. The black line shows the mean plus/minus one standard deviation. Dots represent individual rounds and lines show 5 round averages.

S.7 Questionnaire Results

In the final part of the experiment, subjects must answer a questionnaire which allows us to gain further insights into differences between baseline (B1, B2) and nudging (N1, N2) sessions. Characteristics such as age and gender are not able to explain the differences between baseline and nudging sessions. Subjects have a mean (median) age of 25.27 (25) in baseline sessions and 24.84 (23) in nudging sessions. In total, 48 males and 52 females participated in our experiment with a gender ratio (female/male) of 1.174 in baseline sessions and 1 in nudging sessions.

Personality. The questionnaire contains a set of 30 questions to predict certain personality characteristics of subjects which allows us to examine whether personality can explain differences between baseline and nudging sessions. We selected three personality measures (*Honest-Humility*, *Agreeableness versus Anger*, and *Extraversion*) from the HEXACO-60 inventory that assesses the six dimensions of personality structure of the HEXACO model (31). We find no significant differences in personality between subjects in baseline and nudging sessions, suggesting that differences in personality are not driving our results. For *Agreeableness versus Anger*, we find that the average subject in the baseline sessions (nudging sessions) scores, on a scale from 1 to 5, 3.16 (3.15) which is not significantly different (p-value of a two sample

t-test is 0.94, two sided, *n*=49 in baseline, *n*=50 in nudging).[1] For *Extraversion*, we find that the average subject in baseline sessions (nudging sessions) scores 3.33 (3.5) which is not significantly different (*p*-value of a Welch two sample *t*-test is 0.165, two sided, *n*=49 in baseline, *n*=50 in nudging).[2] For *Honest-Humility* we find that the average subject in baseline sessions (nudging sessions) scores 3.408 (3.398) which is not significantly different (*p*-value of a two sample *t*-test is 0.933, two sided, *n*=49 in baseline, *n*=50 in nudging).

Game Experience. This part of the questionnaire asks subjects about their experiences with the others throughout the game as well as about features of the game itself. The average subject in the baseline sessions (nudging sessions) has 5.15 (5.39) acquaintances and 4.29 (4.57) friends. 56% of subjects reported that their group of friends never changed throughout the game (60% in baseline and 52% in nudging), 29% reported that their group of friends changed every 10 rounds (24% in baseline and 34% in nudging), 13% reported that their group of friends changed faster than every 10 rounds (12% in baseline and 14% in nudging). The average (median) subject reports that it took approximately 13 (13) rounds to find friends in the baseline sessions and approximately 13 (11) rounds in the nudging sessions.[3] In baseline sessions (nudging sessions), 26% (26%) of subjects indicated that they found friends in less than 10 rounds, 44% (40%) needed between 10 and 19 rounds to find friends, 18% (28%) reported that they needed more than 20 rounds to find friends, 8% (6%) indicated that they never found friends, and 4% (0%) are missing.[4]

We asked subjects to score, on a scale from 0 to 10, whether they perceive received inquiries or replies from others as a sign of friendship. The median subject in the baseline sessions (nudging sessions) gives inquiries a score of 5 (6), which is significantly different (*p*-value of a two sample Wilcoxon rank sum test is 0.008, two sided, *n*=48 in baseline, *n*=50 in nudging).[5] The median subject in the baseline sessions (nudging sessions) gives replies a score of 9 (9), which is not significantly different from each other (*p*-value of a two sample Wilcoxon rank sum test is 0.678, two sided, *n*=49 in baseline, *n*=50 in nudging). The results show that

[1] The answer of one subject is missing in the baseline sessions due to a data storage error.

[2] Here we select the Welch two sample *t*-test, because an *f*-test shows that the two sample variances are significantly different from each other (*p*-value of 0.02).

[3] To calculate the mean and median we exclude subjects who reported *never* and treat reports of >25 as 25.

[4] 4% of questionnaire answers (two subjects) are missing in the baseline sessions due to a data storage error.

[5] We choose a non-parametric test because the underlying data in baseline and nudging sessions are not normally distributed. For individual sessions (B1; B2; N1; N2) subjects gave inquiries a mean value of (4.5; 4; 5.5; 5.6) and a median value of (4; 5; 5; 6).

subjects in the baseline and nudging sessions value replies more than inquiries as a sign of friendship. The increase in the value of inquiries in nudging sessions potentially reflects that the networks in nudging sessions generate more profit compared to baseline sessions. We further ask subjects whether they prefer to send inquiries to friends, acquaintances, or neither of both. 75% of subjects indicate that they prefer friends (68% in baseline and 82% in nudging), indicating that inquiries carry a strong friendship signal. 73% out of the 75% of subjects who prefer friends also indicate that they prefer some friends over others when sending an inquiry. Replies, however, tell a different story. 59% of subjects (58% in baseline and 60% in nudging) indicate that they have no preference over sending replies. That is, the majority of subjects reply independently of whether they consider the individual they are about to help as a friend or an acquaintance. Out of the 33% (30% in baseline and 36% in nudging) who prioritize friends as the receiver of a reply, 61% report that they have no preferences among their friends.

General Questions. We asked subjects whether the game is good at mimicking actual communication, e.g., email communication in an actual business, on a scale from 1 to 10. The average (median) subject gives our game a score of 5.6 (6). Furthermore, our game features a timer that ends a round if the last three subjects need more than 15 seconds to make their decision to prevent the game from stalling. We were worried that this might influence the decisions of subjects so we asked them whether the timer influenced their decisions in the game. 90% of subjects indicated that the timer did not (52%) or just marginally (38%) affect their decisions, confirming that the timer is not responsible for the outcome of the game. Lastly, we asked subjects to indicate whether they were surprised that the game ended since we set a time limit of 90 minutes instead of letting them play a fixed number of rounds. 90% of subjects indicated that they were not surprised (52%) or a little surprised (38%), showing that there might be an endgame effect in the last rounds of our game. However, the data does not confirm such an endgame effect.

S.8 Experimental Instructions

<u>Game Instructions (Note that these Instructions resemble the Tutorial)</u>

Please read through this tutorial carefully to understand the rules of this experiment. Note that at the end of the tutorial you must answer some questions. You can proceed with the experiment only after having correctly answered all the questions. It is important that you understand the "Game" since it is likely that you earn more money when you understand the rules.

Payment

In this experiment, you can earn ECU (Experimental Currency Unit) that will be converted into DKK (Danish krone) at the end of the experiment. The exchange rate is 1:3, which means that for each ECU you have after the experiment, you will receive 3 DKK.

At the end of the experiment you will be paid what your balance is plus 50 DKK as a show-up fee. For example, if your balance is 150 ECU at the end of the game you receive 500 DKK (3x150 + 50).

If you leave before the Experiment is finished you will not receive any money.

Objective

This experiment consists of a series of rounds. In the beginning of each round (stage 1) you will be assigned a "**Question**" and an "**Expertise**".

Your assigned "**Question**" is indicated by a letter (e.g. A, B, C ....). For example, if you are assigned with question A, your goal is to find the player who has the corresponding expertise (In this case a player with expertise A).

Your "**Expertise**" is indicated by a different letter (e.g. if your question is A it is not possible that your expertise is A). For example, if you are assigned with expertise B, some other player assigned with question B is searching for you.

During each round, your goal is to find out which of the other players is the "**Expert**" for your "**Question**". If you succeed, you will earn 10 ECU.

How to play

You communicate with the other players by sending and receiving messages. It costs 1 ECU to send a message.

Each round has 2 stages:

- In the first stage, you can send "**Inquiries**" which reveal your question and expertise to the receiver, and ask for help finding your expert. If multiple players send inquiries to you, you might learn that one is the expert another player is searching for.
- In the second stage, you can "**Reply**" to the inquiries you received from the first stage. If you reply to an inquiry, and you know who the expert is, you help the receiver fulfill her task.

In each stage, you may send messages to as many players as you want (but it costs 1 ECU to send each message). The messages will be delivered simultaneously at the end of each stage when all players are finished sending messages.

In the following, you will be guided through an example, which teaches you the game interface and its functions.

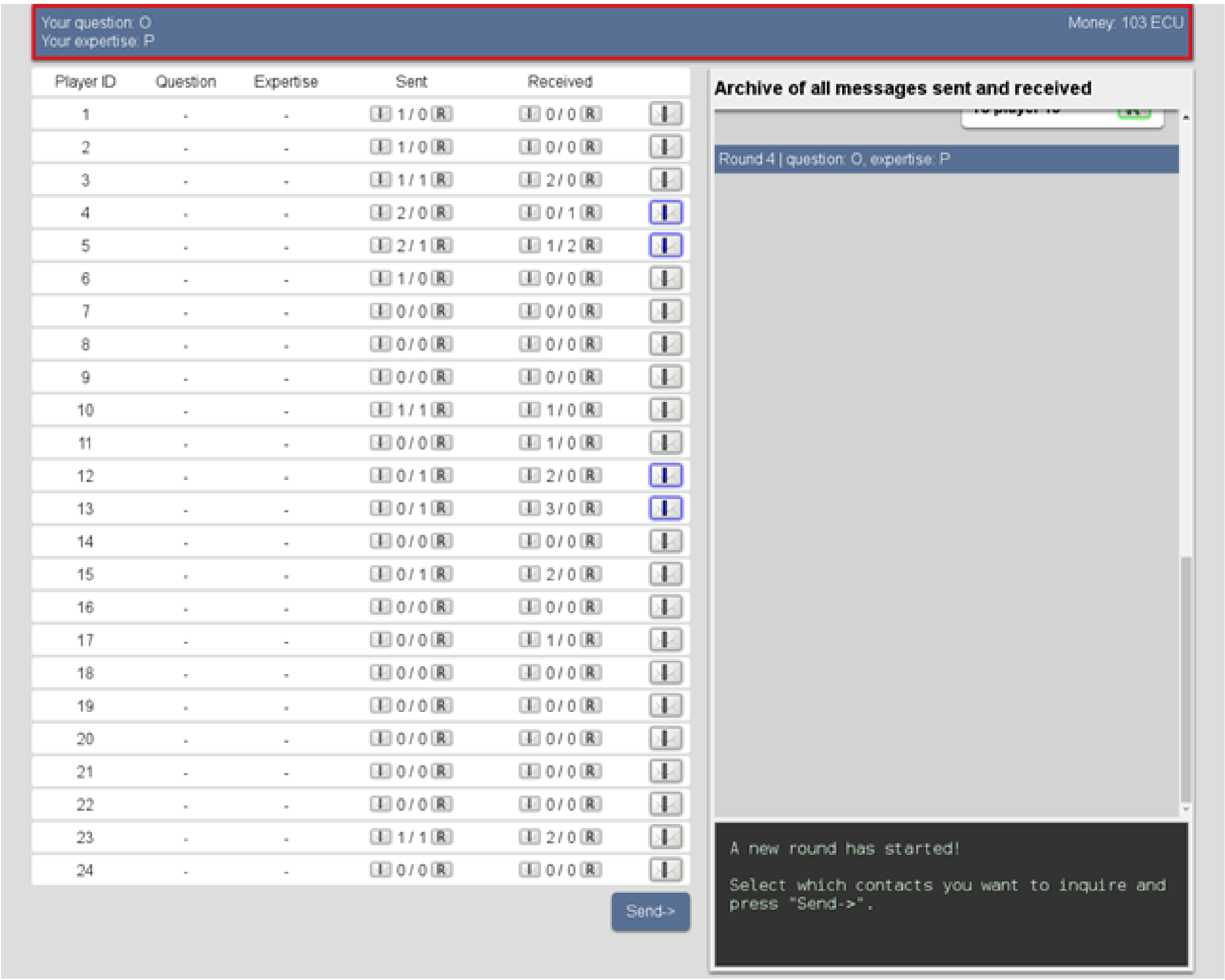

At the top of the screen you can find information about your current state in the game.

- To the left you will find your own "**Question**" and "**Expertise**".
- To the right you can see how much ECU you currently have. When the experiment begins you have 100 ECU.

In this example, your current "**Question**" is "**O**" and your "**Expertise**" is "**P**". This means that during this round you have to find the player with expertise "**O**" to earn 10 ECU. The player with question "**P**" has to find you to earn 10 ECU for himself.

In the example you have 103 ECU, in the beginning of the fourth round. This means that during the first three rounds, you have won 3 ECU more by finding your expert, than you have spent sending messages.

Notice that the panel on top of the screen is **blue** whenever you are expected to make an action, and **grey** when you are waiting for the other players. A *bell*-sound will be played whenever a new stage begins, i.e. when the top-panel changes color from **grey** to **blue**.

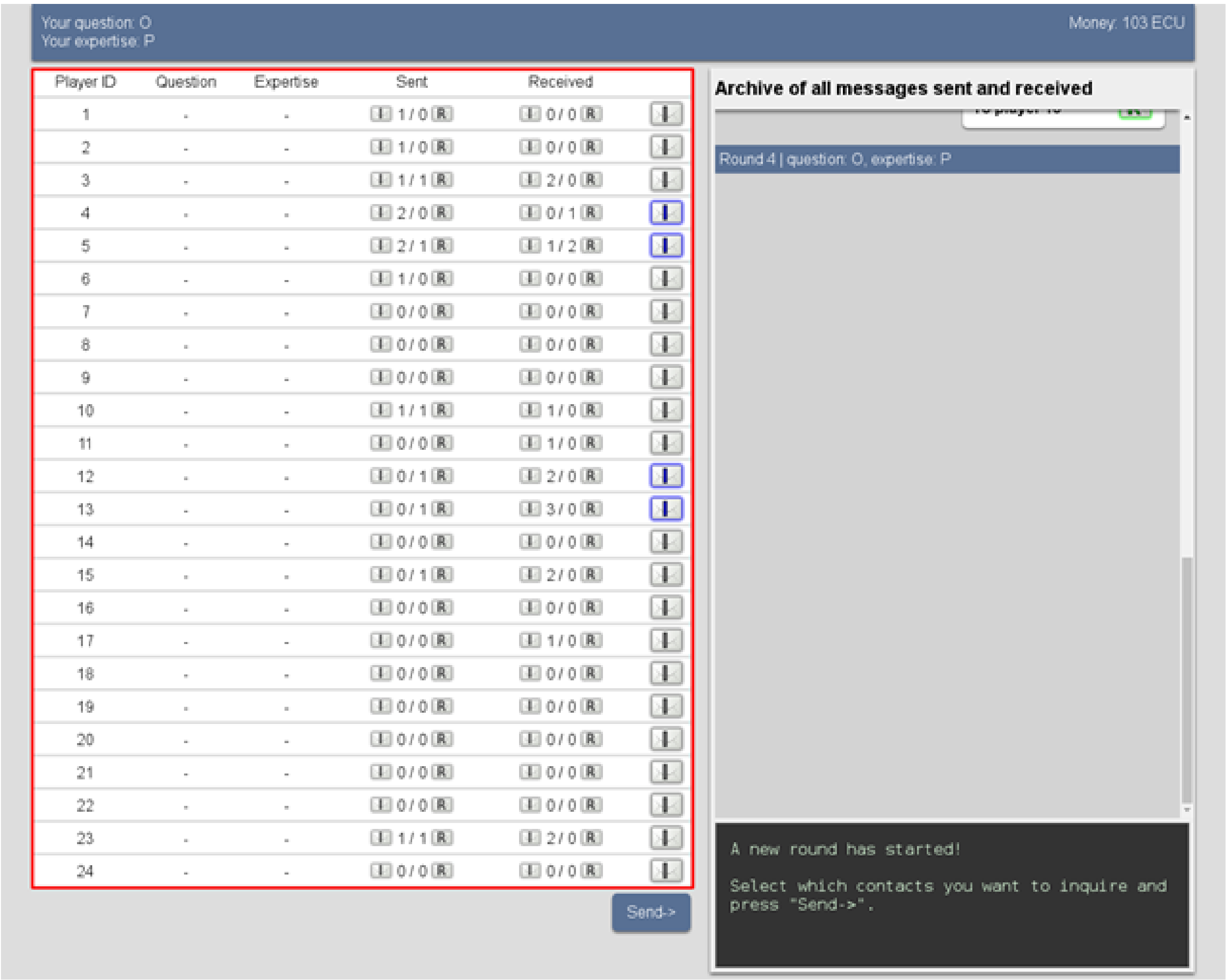

To the left you will find the ID of all other players (from 1- 24) and information about their "**Question**" and "**Expertise**" in the current round. Note that the number of other players in the session today might be different from this example.

Important: Question and expertise will change at the beginning of each round but the player ID will remain fixed throughout the entire experiment. This means that your ID and the other players ID will not change (e.g. player 17 will be player 17 through the entire experiment). The only thing that changes between rounds is your and other players "**Question**" and "**Expertise**".

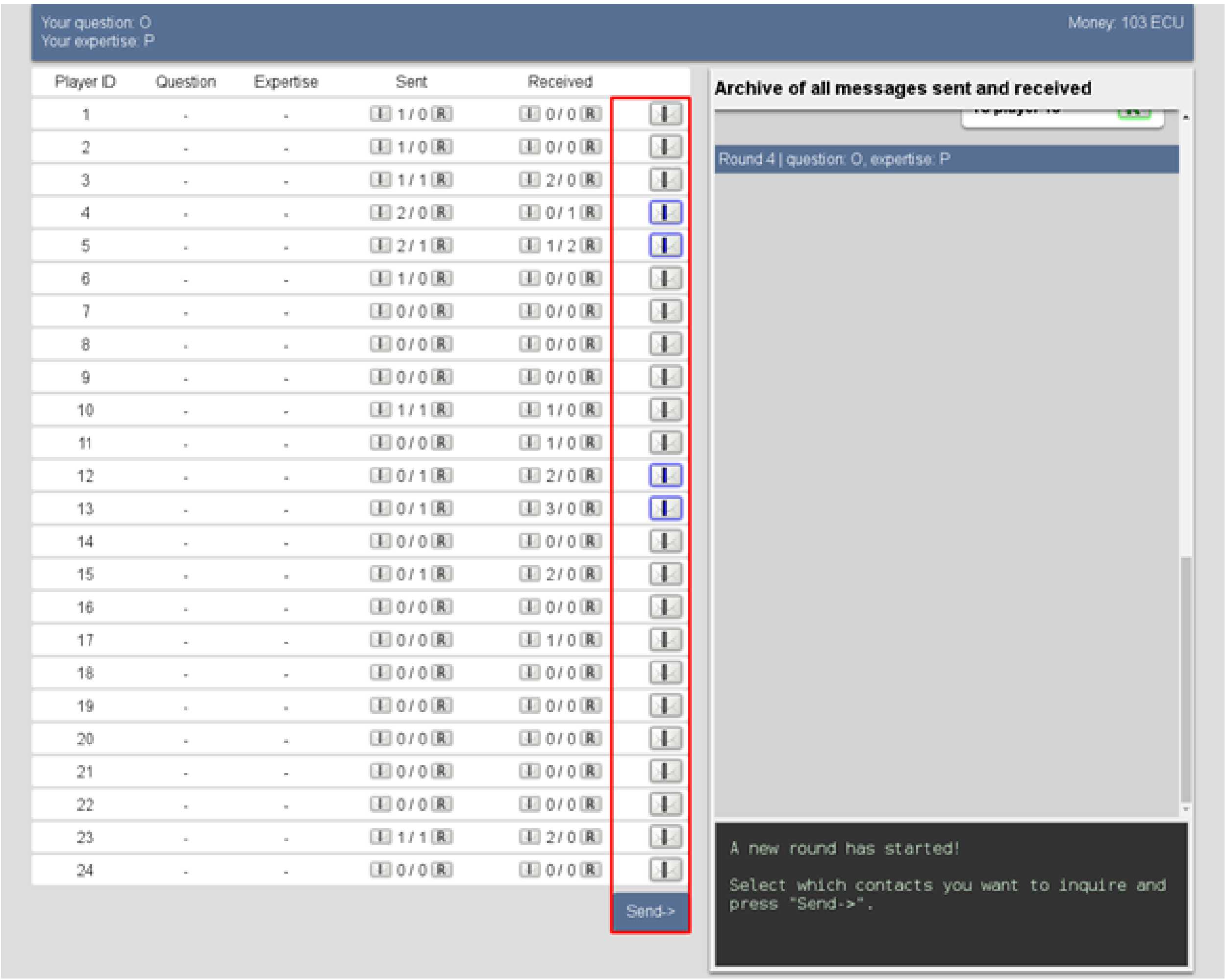

In stage 1 you can send "**Inquiries**" to the other players.

To select who you will send "**Inquiries**" to you click the inquiry-icon 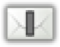 in the right end of each player label. When clicked, the color of the icon will change from grey to blue 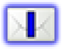 indicating that the player is selected. In the example, you have selected players 4, 5, 12 and 13 (Note that it is possible to undo your choice i.e. you can press on a blue icon and it turns grey again).

Sending an "**Inquiry**" costs 1 ECU. Remember, you must pay this cost for each inquiry you send. For instance, if you send your inquiry to 4 different players you must pay 4 ECU.

Once you have made your choice you must press the "**Send->**"-button to send your inquiry to the selected players (i.e. player with a blue icon 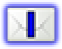).

When you have sent your inquiries, you have to wait for the other players to send theirs too. All inquiries will be delivered simultaneously after all players have clicked "**Send->**".

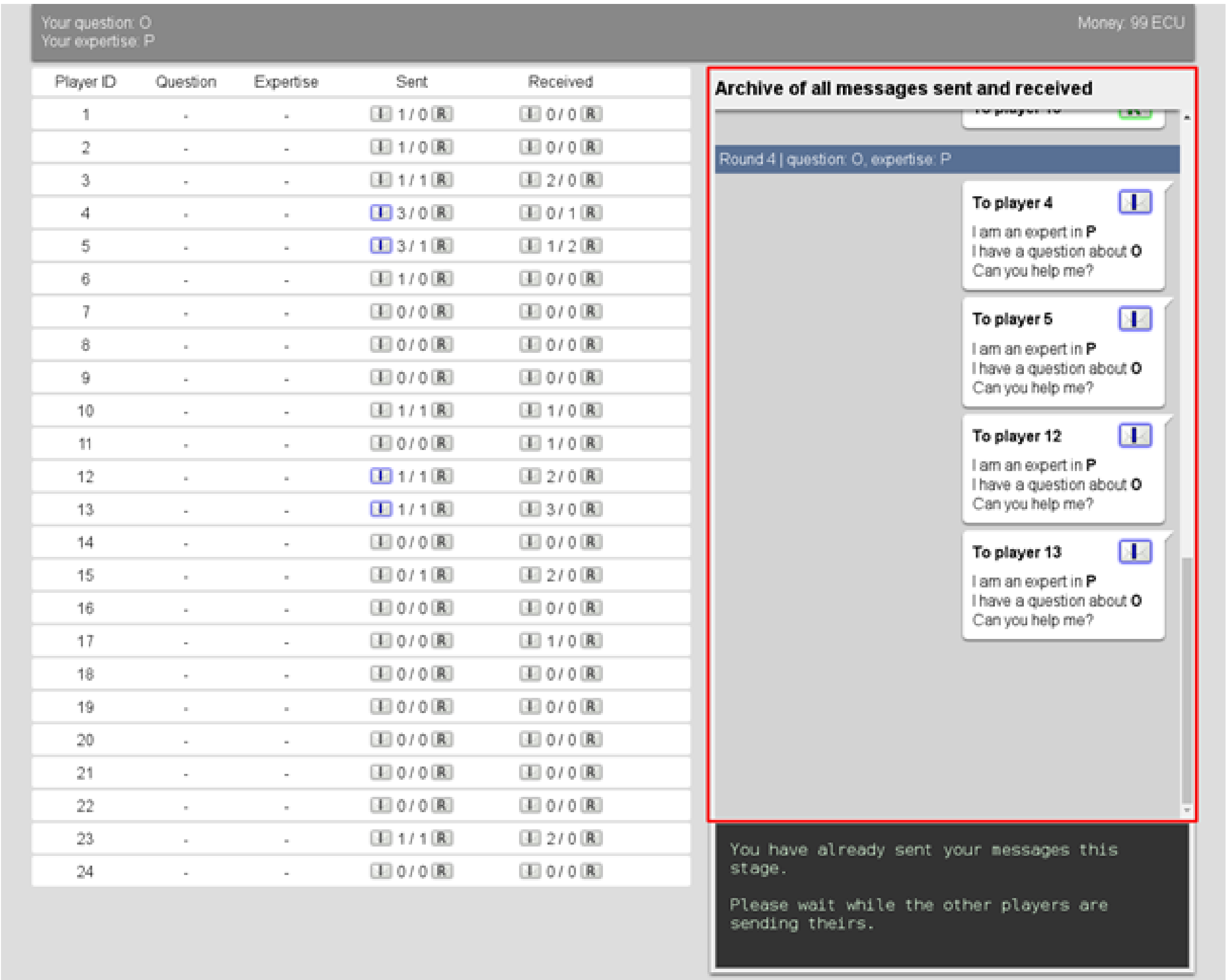

Here you can see the messages you sent and received during the entire experiment.

In the example, in the current round (round 4) you have sent "**Inquiries**" to players 4,5,12, and 13, and you are now waiting for the other players to send their messages, before the game can continue. Notice that the top panel is grey because you are waiting for the other players.

To see the messages you sent and received in the previous rounds you can scroll in the "Archive of all messages sent and received".

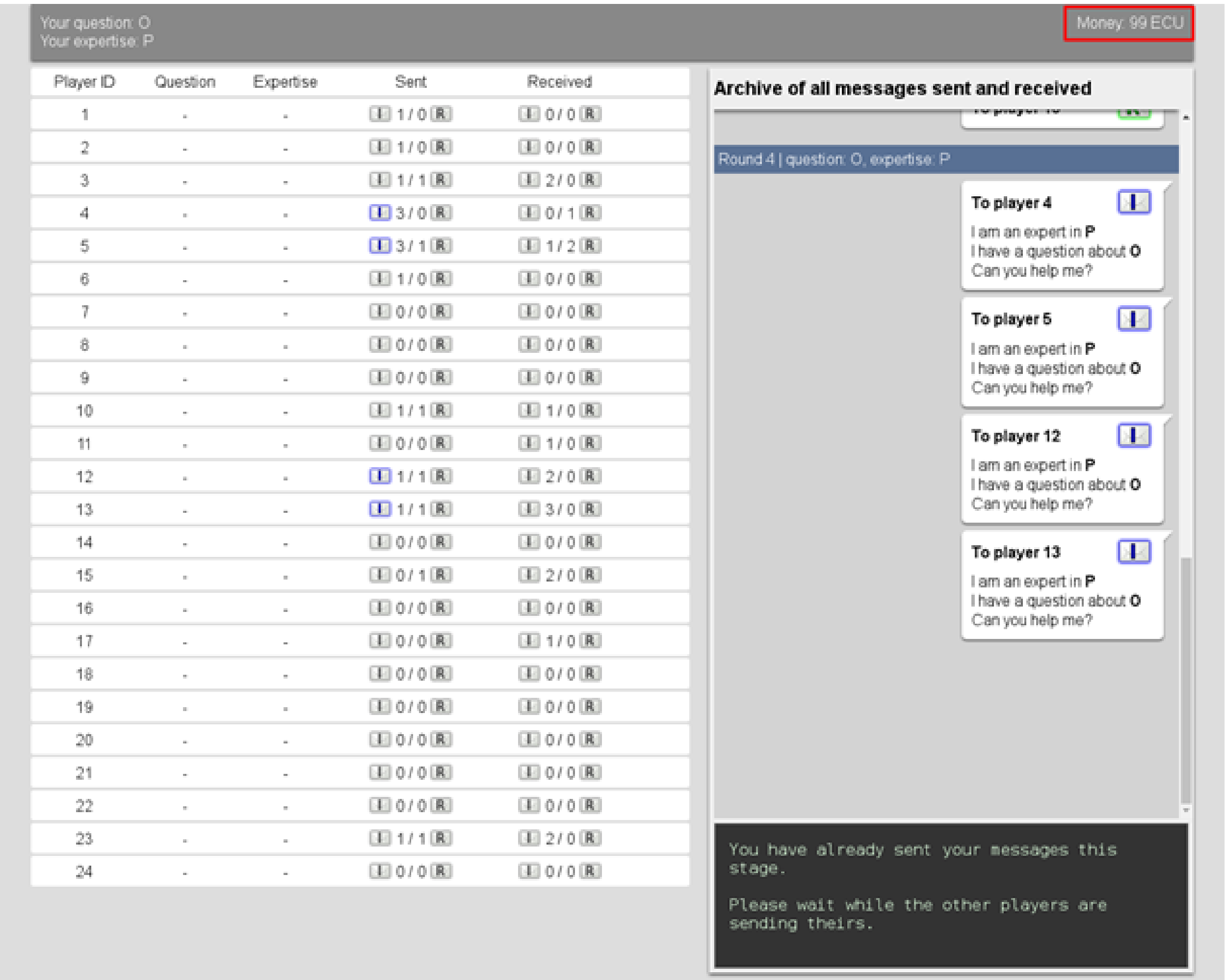

In the example you spent 4 ECU for sending 4 inquiries. The balance in the upper part of the screen is now 99 ECU (103 – 4 ECU).

Note that it is not possible to send messages, if their combined price is higher than your current balance.

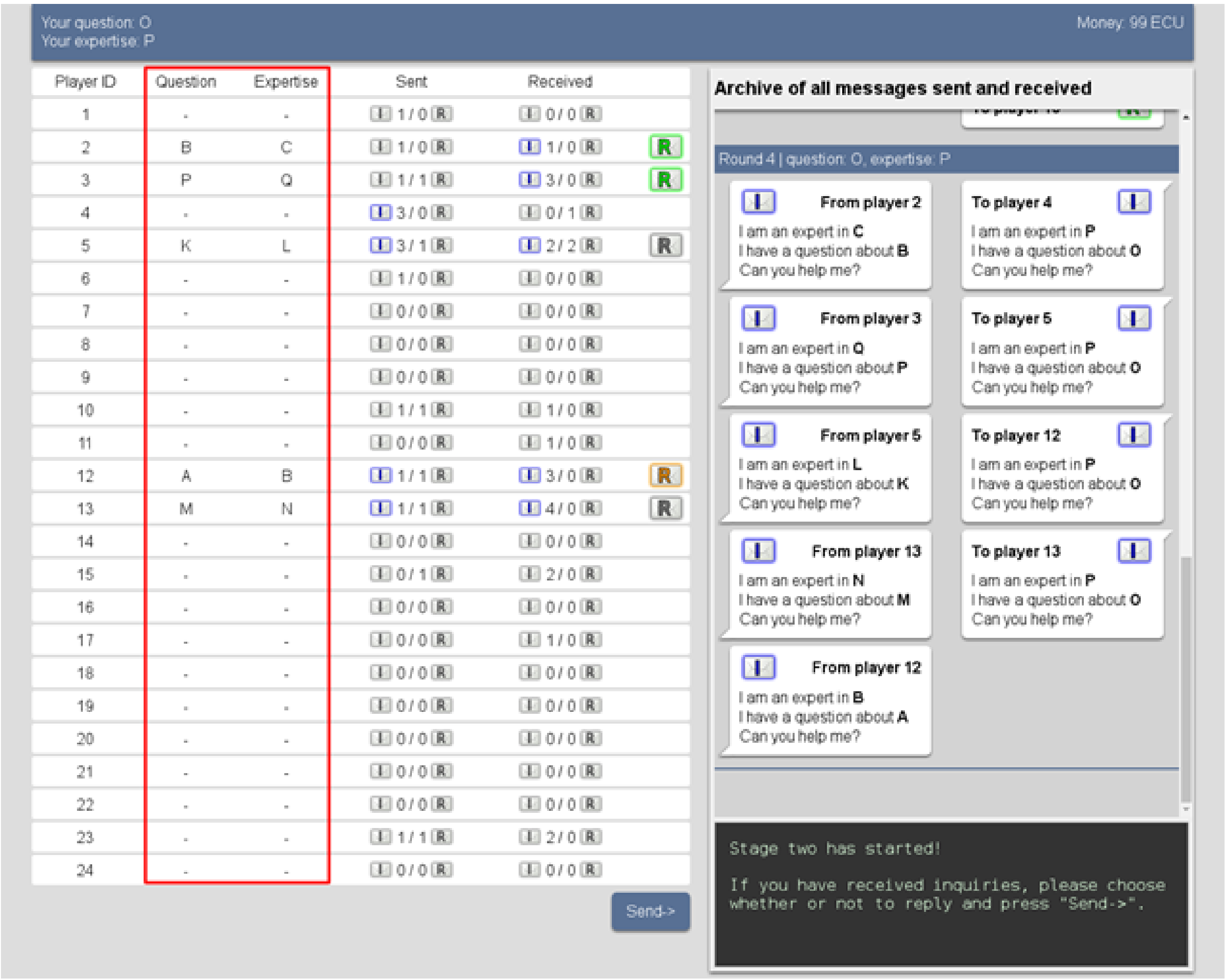

When the last player has clicked send, you will receive the "Inquiries" that other players have sent to you.

When you receive an inquiry from another Player, her "**Expertise**" and her "**Question**" will appear in the player table.

In the example, you received inquiries from players 2, 3, 5, 12, and 13. Now you know, for example, that in the current round player 2 has a question "**B**" and is an expert in "**C**".

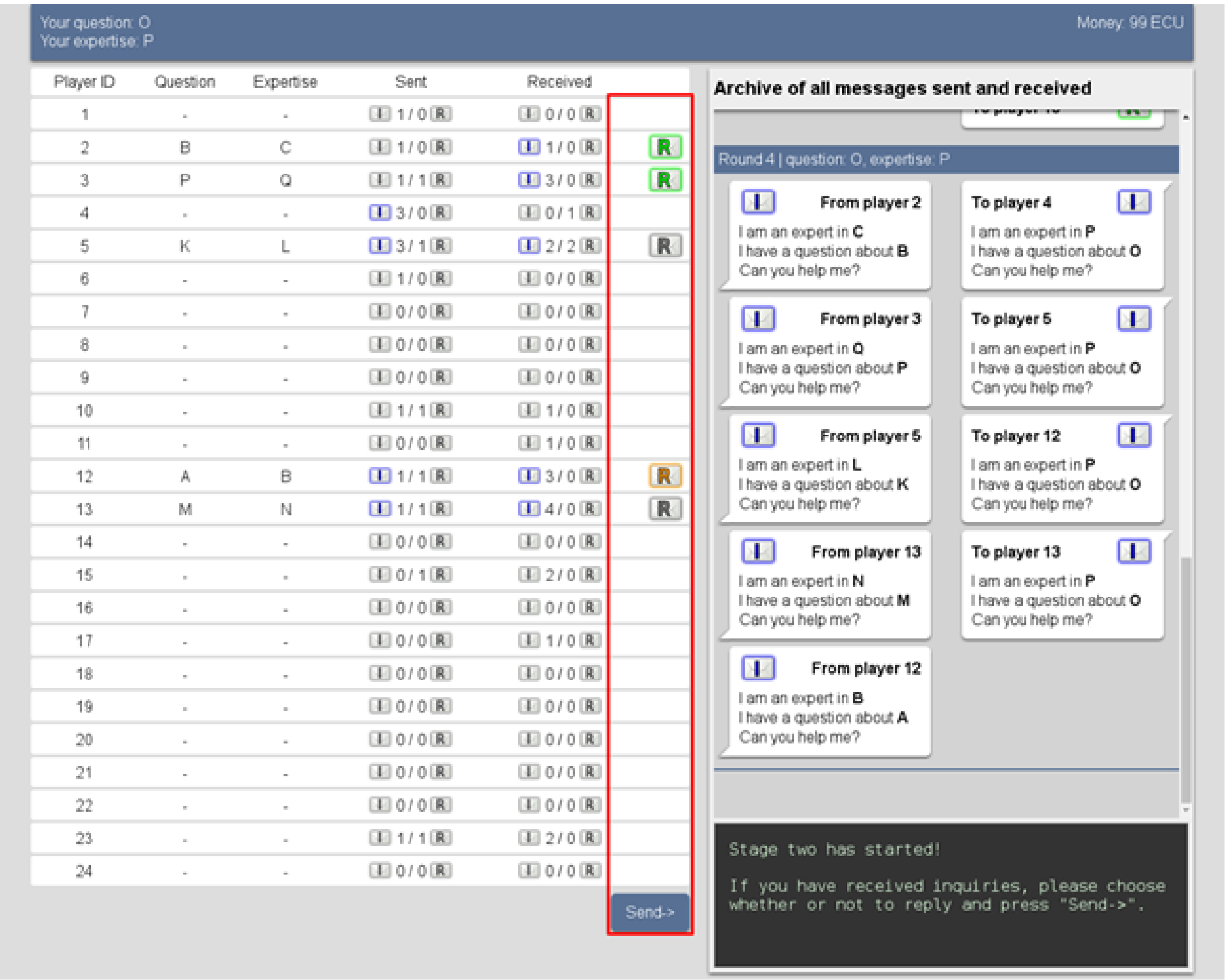

Stage 2 starts immediately after the inquiries sent in stage 1 are delivered. Note that there are now reply icons 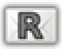 to the right of the players table. An icon will appear only next to the players from whom you received inquiries. In the example, that are players 2, 3, 5, 12, and 13.

In this stage, if you want, you can reply to the other players' inquiries. Sending a reply can help the receiver find her expert. You may reply to any inquiry you have received. Each reply costs 1 ECU.

You can send two types of (automatically generated) replies:

1. If you know who has an "**Expertise**" matching the inquirer's "**Question**", your reply will say: The expert you are looking for is "Experts player ID", and the reply symbol will be green .

2. If you do not know it, it will say: I'm sorry, but I don't know anyone who's an expert for this "**Question**", and the reply symbol will be orange .

You select which players to reply to by pressing grey reply icons in the right end of the player-labels. A player is selected when the icon changes color. Then press "**Send->**" to actually send the replies.

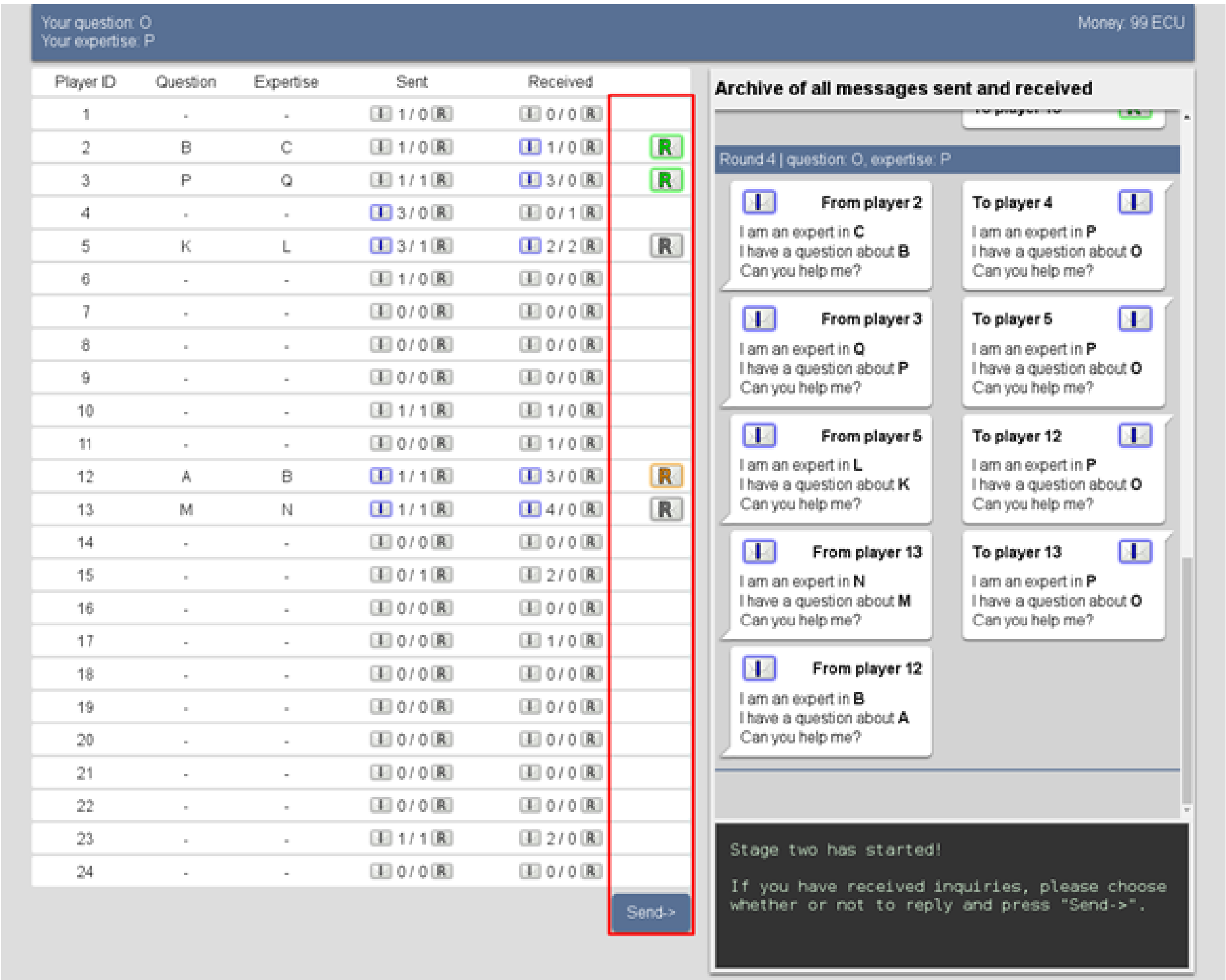

In this example, you are about to send 3 replies (to players 2, 3, and 12) which will cost 3 ECU in total (3 x 1 ECU). You can send as many or as few replies as you want.

- The reply to player 2 is green, because player 2 has a question about "**B**" you know that player 12 is the expert in "**B**". The reply text will be: "The expert you are looking for is player 12".
- The reply to player 3 is also green, because player 3 has a question about "**P**" and you are an expert in "**P**". The reply text will be: "Yes, I happen to be the expert you are looking for".
- The reply to player 12 is orange because player 12 has a question about "**A**", but you don't know who is an expert in "**A**". The reply text will be: "I'm sorry, but I don't know anyone who's an expert in **A**".

In the example, the reply icons 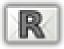

of player 5 and 13 stay grey, because you choose not to reply to them.

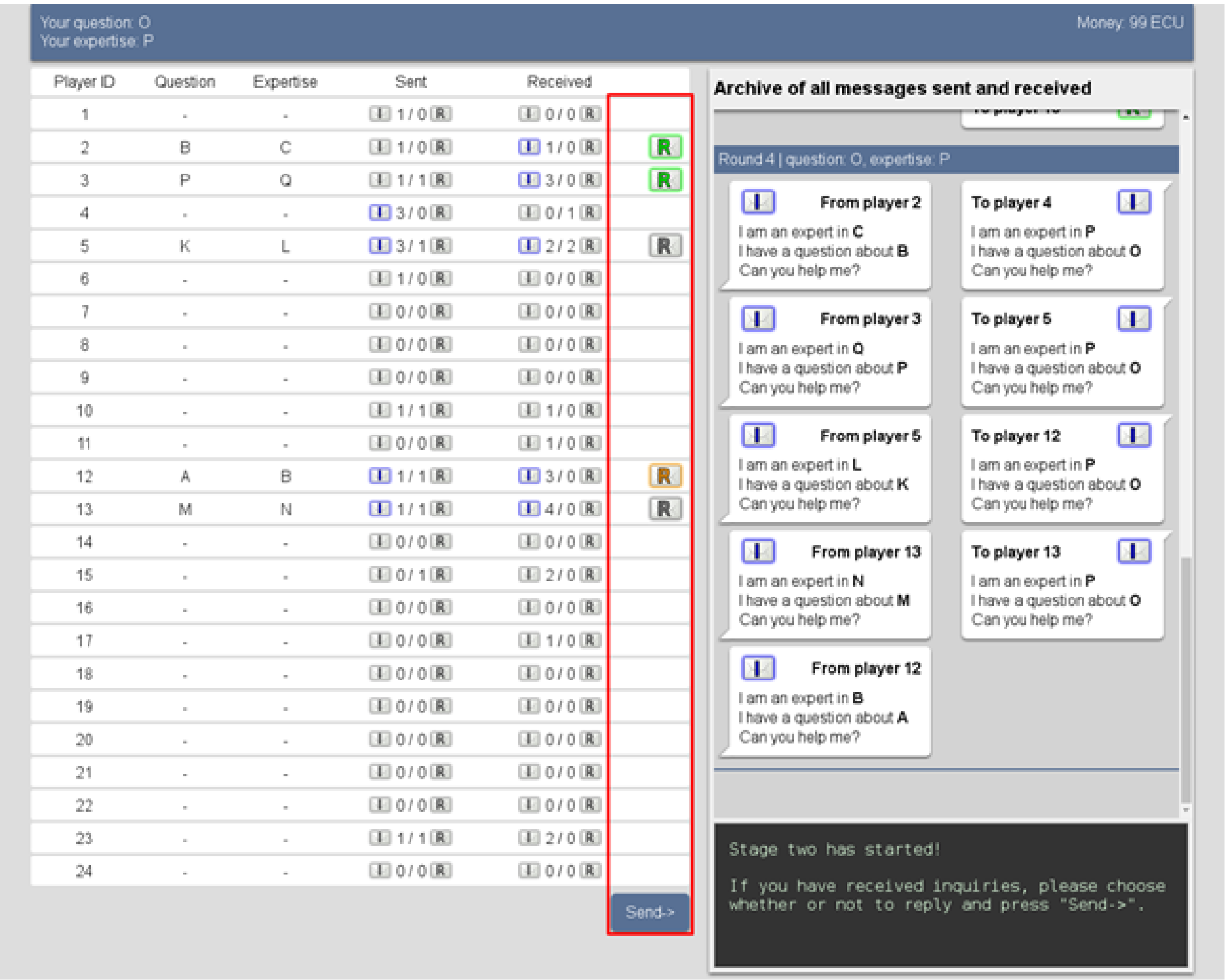

Note that you cannot send false information.

For instance, you cannot send an orange reply (type 2) to player 2. Since you know who the expert of player 2 is, your only option is to either send a green reply (type 1) or you choose not to reply.

In addition, it is not possible to send a green reply (type 1) to player 12. Since you do not know who his expert is, your only option is to either send an orange reply (type 2) or you choose not to reply.

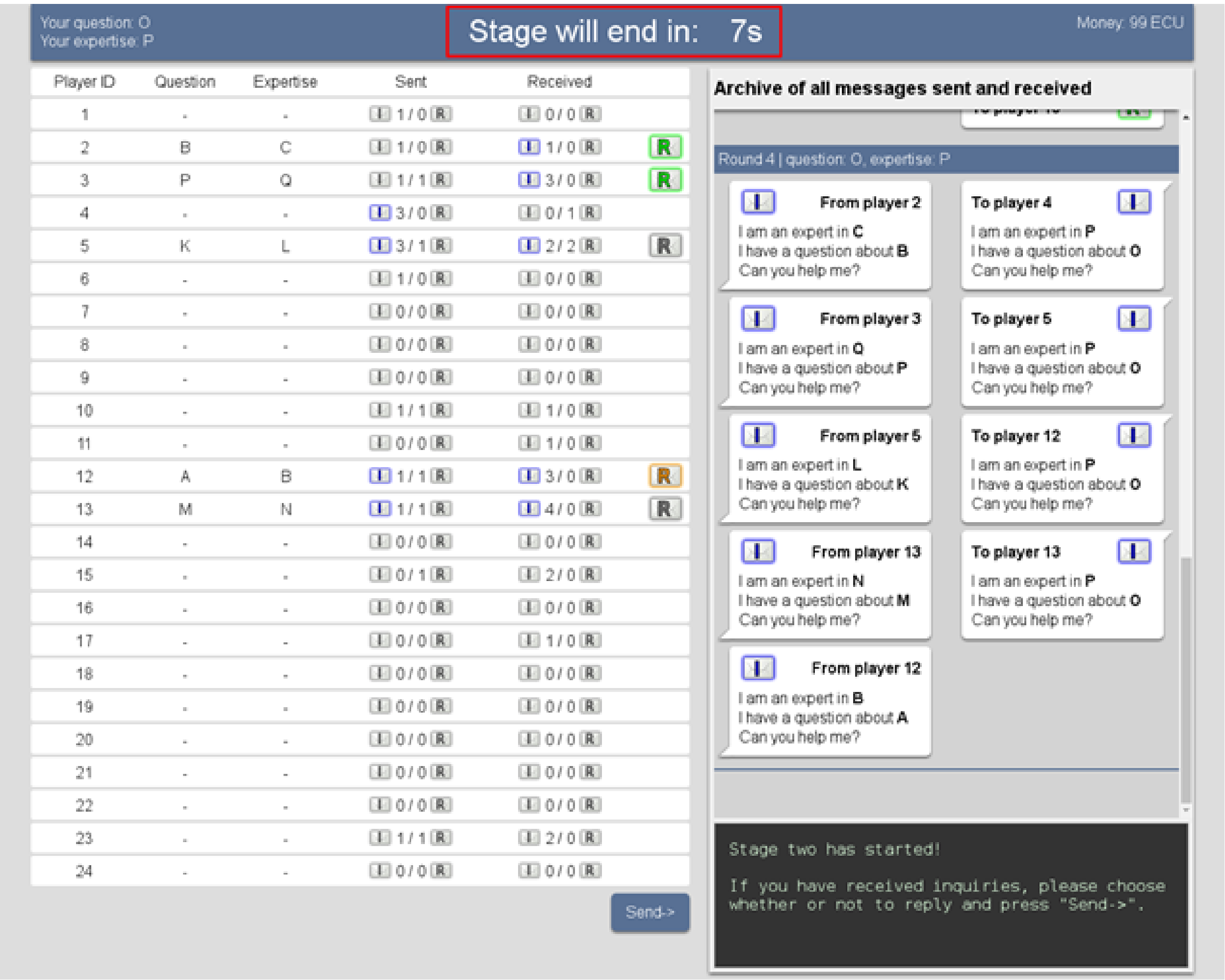

Each stage lasts until the last player has clicked the "**Send->**" button. To prevent the experiment from stalling, the game implements a "soft timer".

The "soft timer" works in the following way:

When all players except for 3 have sent their messages, this timer will appear at the top of the screen of the last three players. The timer will start at 15 seconds, and count down to 0 seconds.

If you are one of the last three players and you don't click the "**Send->**" button before the timer reaches zero seconds, the game will automatically click the button for you. An automatic click works exactly like a manual click, so if the player has selected players to send messages, these messages will be sent.

In the example, you are one of the last three players who have not yet clicked the "**Send->**" button in the current stage, so the timer has started. There are currently 7 seconds until the game will automatically click the send button. If that happens, you will send green replies to the players 2 and 3, an orange reply to player 12, and no replies to player 5 and 13.

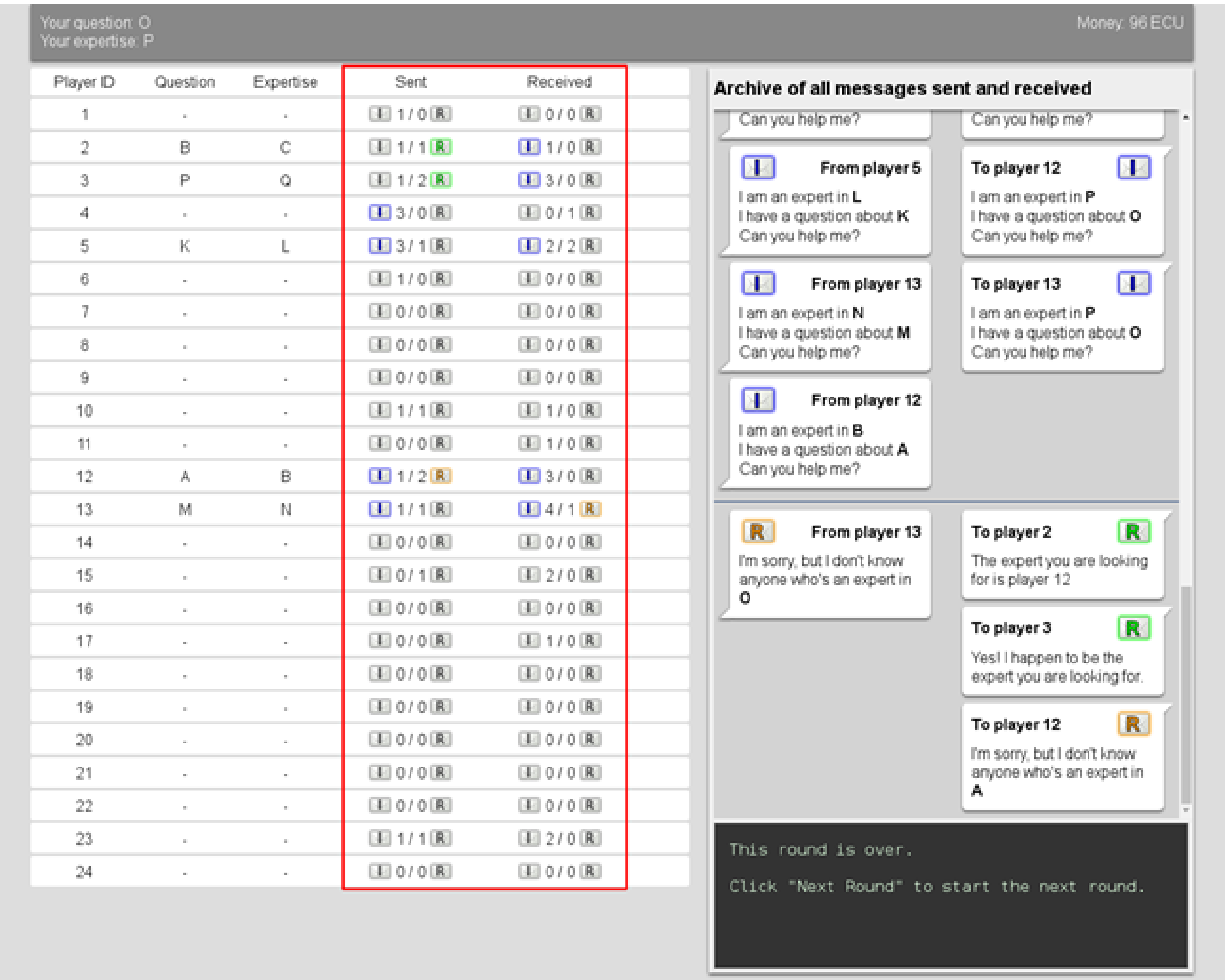

You can use the **message-counters** to get a fast overview of the messages you sent and received in the game.

The color of the small message icons (inquiry: 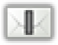 and reply: 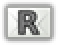) show whether you have sent or received such a message in the current round.

- **Sent Column:** In the example you can see, that in the current round you have sent inquiries to the players 4, 5, 12, and 13, and you have sent green replies to the players 2 and 3, and an orange reply to player 12.
- **Received Column:** You can also see that you received inquiries from the players 2, 3, 5, 12, and 13, and you received an orange reply from player 13.

The numbers show how many messages you have sent and received, of the corresponding type, during the entire experiment.

- E.g. in the example your entire correspondence with player 5 consists of you sending 3 inquiries (including one in the current round) and one reply, and you receiving 2 inquiries (including one in the current round) and 2 replies.

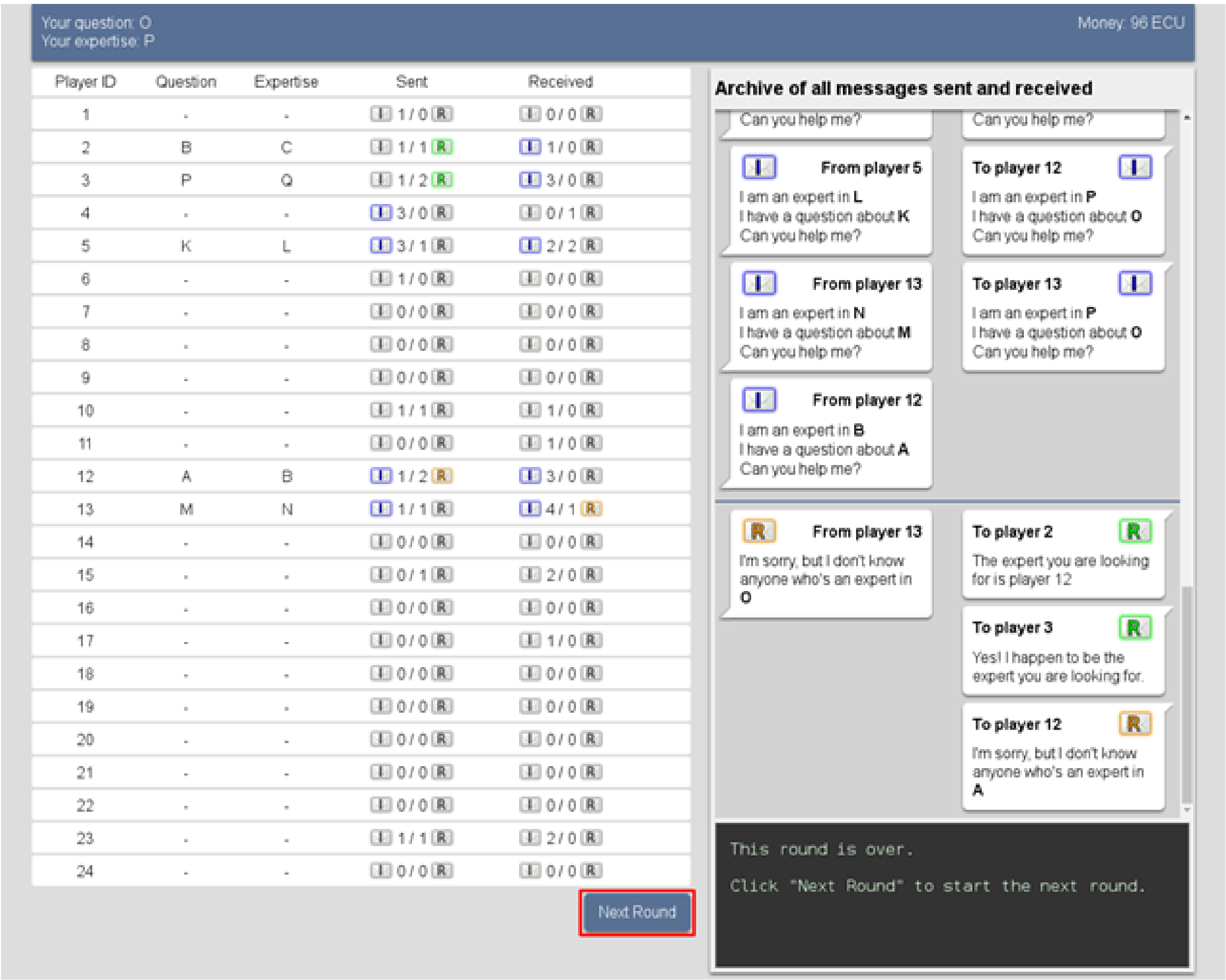

When the last player has sent her or his replies by clicking the "**send->**" button, all replies are delivered and the round is finished.

In the example, you did not find the player who is an expert in "**O**" (your Question). Therefore, you did not earn 10 ECU this round.

When you are finished reading the replies you have received you can start the next round by clicking the "**Next Round**" button.

The experiment consists of many rounds. After 90 minutes, the last round will be allowed to finish, but no new round will be started. You will notice this automatically as the "Next Round" button will not appear on the screen.

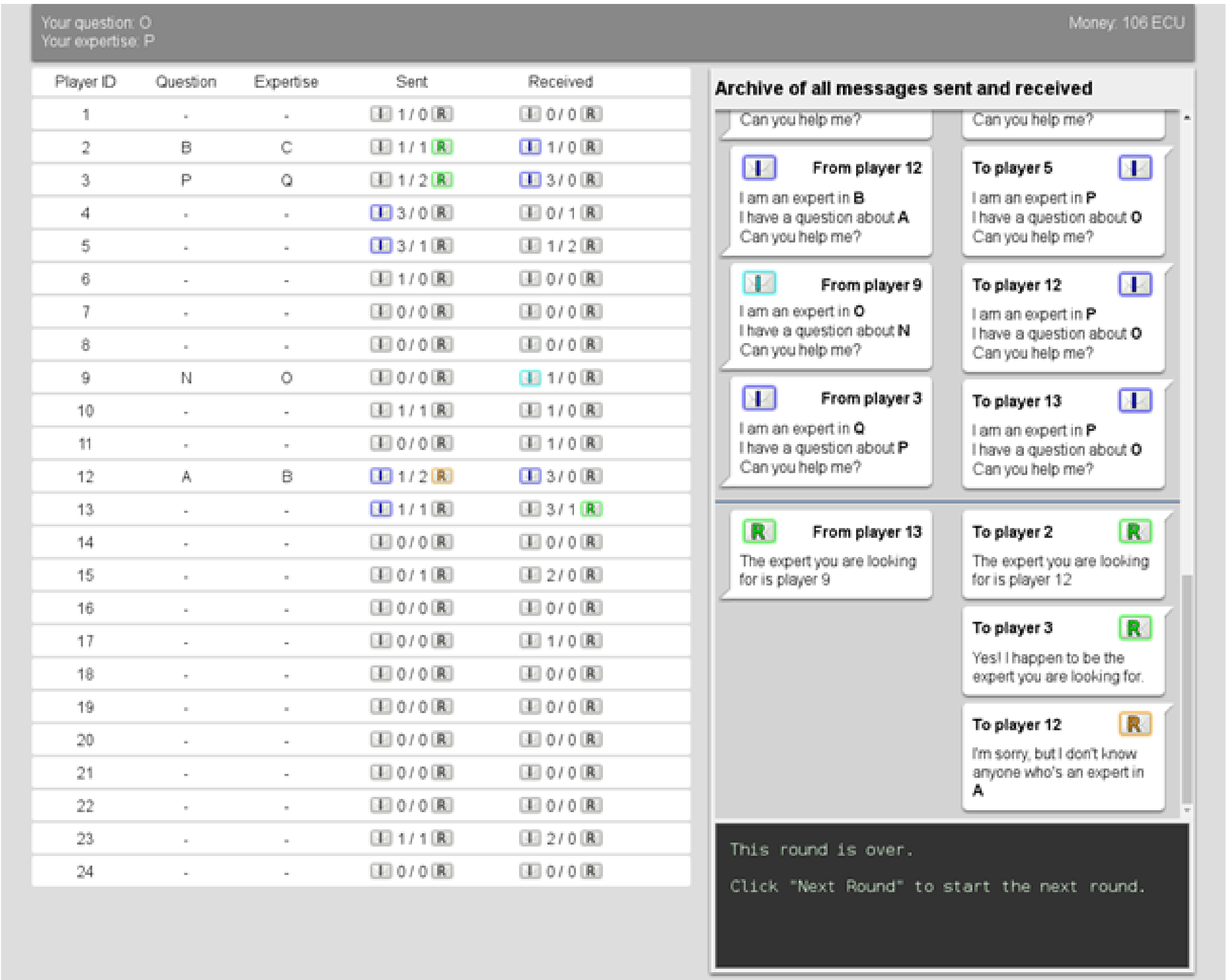

This example shows an altered version of how the round could have played out.

In contrast to the previous example, you have here received an inquiry from player 9. Player 9 is an expert in "**O**" and you have a question about "**O**". The inquiry from player 9 is marked with a turquoise symbol, to indicate that it came from your expert.

In this example, the reply you received from player 13 is green, and is telling you that player 9 is the expert you were looking for. Player 13 must have gotten an inquiry from player 9, to be able to send you this reply.

NOTICE that in this round you have found your expert and won 10 ECU already when receiving the inquiry from player 9 in stage 1. When you received the reply from player 13, you did not win 10 ECU again, because you already knew who your expert was. It is never possible to learn who your expert is and win 10 ECU more than one time each round.

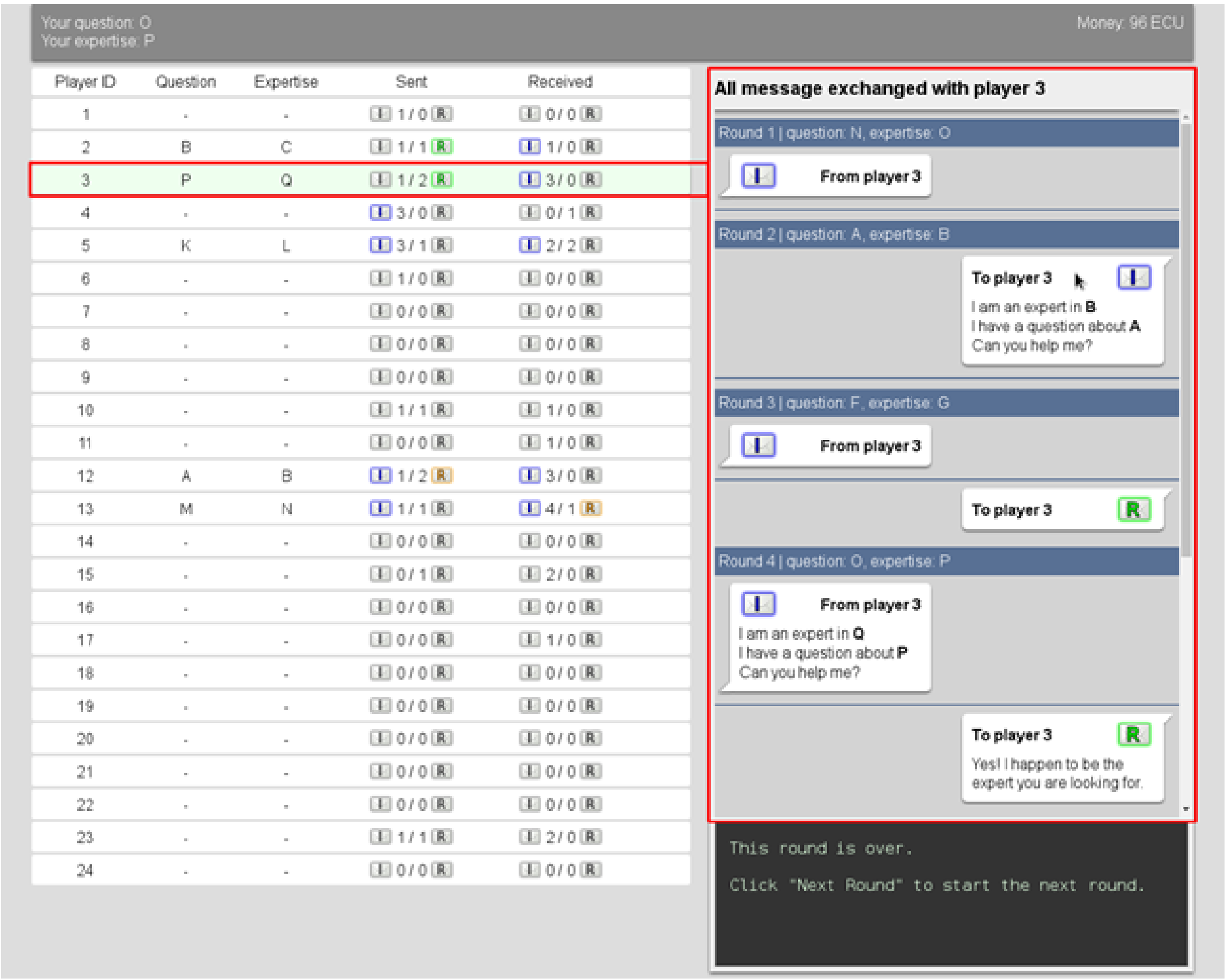

Additional Features

If you click one of the player labels, the archive of messages will show the messages you have sent to-, and received from this player only. You can use this if you want to have a closer look at your correspondence with this particular player.

The archive of messages will return to showing all messages when you click anything that is not a player-label (including the background).

In the example, you have clicked the label of player 3.

Notice that messages you sent and received in previous rounds only display who the message was to or from, and an icon indicating what kind of message it was. To see the content of a message, hold the cursor above it (as with the inquiry sent to player 3 in round 2 in the example).

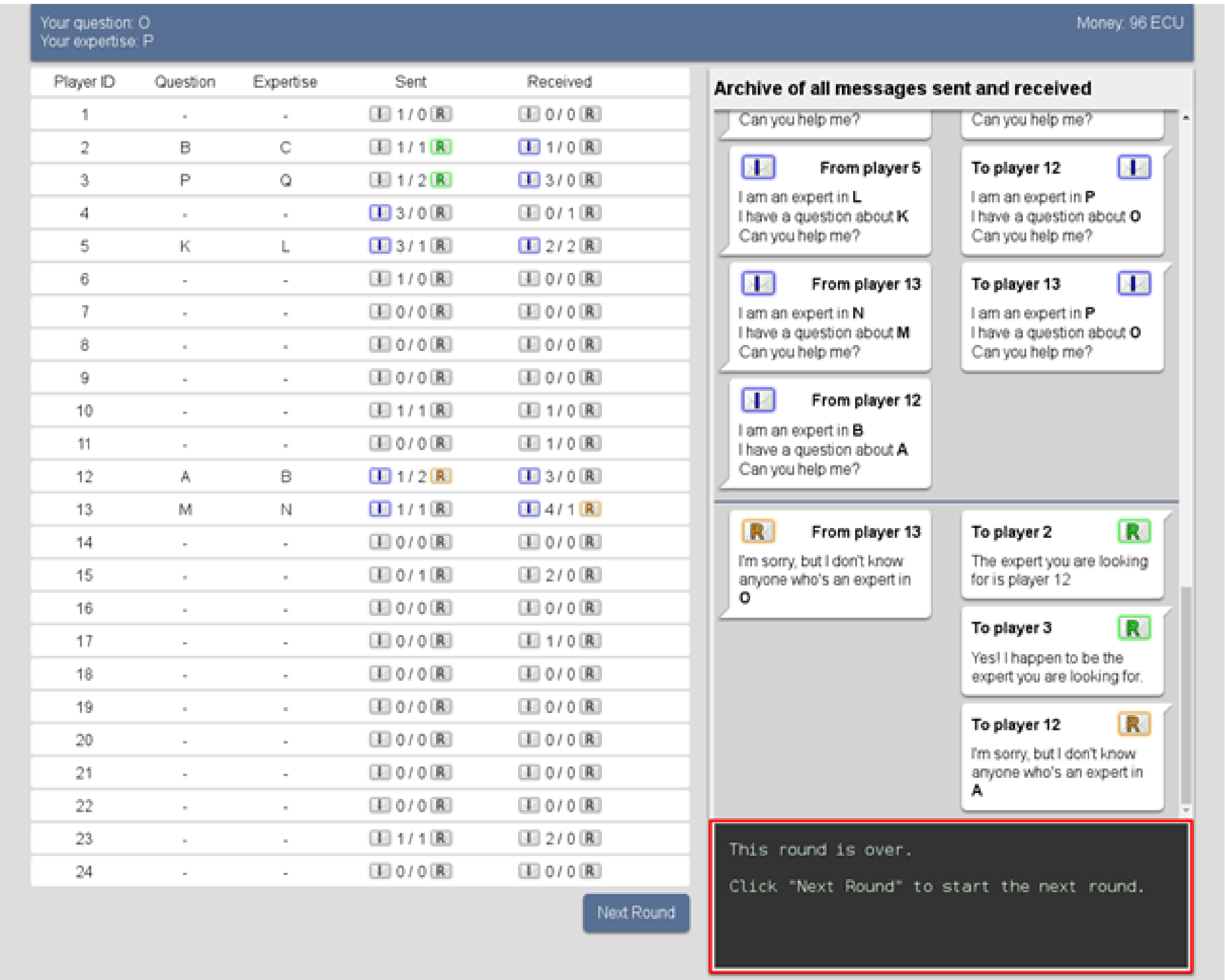

Additional Features

In the bottom right corner of the screen you can see a dialogue window. If you are ever in doubt about what to do during the game, you are encouraged to look here for instructions of what to do next.

If you have any questions please raise your hand and one of the lab-assistants will assist you in private. Do not talk, communicate or ask questions to the other participants. Thank you!

<u>Suggestion Screen</u>

Important!

For the first 5 rounds, six stars will appear next to six specific players. These stars suggest you six other players to contact. We believe this suggestion is beneficial for you and other players. Following this suggestion can help you find your expert and thus collect more ECU.

Note that:

- Each player receives personalized suggestions. This means that all the players have different suggestions of players to contact.
- The suggested contacts are reciprocal. That means that you are a suggested contact for the same players who are suggested contacts for you.
- If every player follows our suggestion and helps other players find their expert, then every player will find his expert in every round.
- You are free to follow our suggestion or contact any of the other players.
- Stars will appear only for 5 rounds. Note that in these five rounds stars will always suggest the same other players.
